# Qualitative and quantitative ethnobotanical study of the Pangkhua community in Bilaichari Upazilla, Rangamati District, Bangladesh

**DOI:** 10.1186/s13002-019-0287-2

**Published:** 2019-02-05

**Authors:** Mohammad Omar Faruque, Gang Feng, Md Nurul Amin Khan, James W. Barlow, Umme Ruman Ankhi, Sheng Hu, M. Kamaruzzaman, Shaikh Bokhtear Uddin, Xuebo Hu

**Affiliations:** 1 Ethnobotany and Pharmacognosy Lab, Department of Botany, University of Chittagong, Chittagong, 4331 Bangladesh; 2 Laboratory of Drug Discovery and Molecular Engineering, Department of Medicinal Plants, College of Plant Science and Technology, Huazhong Agricultural University, Wuhan, 430070 China; 3 National-Regional Joint Engineering Research Center in Hubei for Medicinal Plant Breeding and Cultivation, Wuhan, 430070 China; 4 Medicinal Plant Engineering Research Center of Hubei Province, Huazhong Agricultural University, Wuhan, China; 5 Wuhan Fourth Hospital, Puai Hospital, Tongji Medical College, Huazhong University of Science and Technology, Wuhan, 430034 China; 6 Kurigram Government College, Kurigram, Bangladesh; 7 Department of Chemistry, Royal College of Surgeons in Ireland, Dublin, Ireland; 8 Hubei Cancer Hospital, Wuhan, 430034 China; 9 The State Key Laboratory of Agricultural Microbiology, Ministry of Education, Department of Plant Pathology, Collage of Plant Science and Technology, Huazhong Agricultural University, Wuhan, Hubei China

**Keywords:** Indigenous community, Traditional healer, Ethnomedicine, Ethnobotany, Bangladesh

## Abstract

**Background:**

The present study documents the ethnomedicinal knowledge among the traditional healers of the Pangkhua indigenous community of Bangladesh. The documented data from this area was quantitatively analyzed for the first time. We aimed to record ethnomedicinal information from both the traditional healers and also the elderly men and women of the community, in order to compile and document all available information concerning plant use and preserve it for the coming generations. We aimed to compare how already known species are used compared to elsewhere and particularly to highlight new ethnomedicinal plant species alongside their therapeutic use(s).

**Methods:**

All ethnomedicinal information was collected following established techniques. Open-ended and semi-structured techniques were primarily utilized. Data was analyzed using different quantitative indices. The level of homogeneity between information provided by different informants was calculated using the Informant Consensus Factor. All recorded plant species are presented in tabular format, alongside corresponding ethnomedicinal usage information.

**Results:**

This investigation revealed the traditional use of 117 plant species, distributed among 104 genera and belonging to 54 families. There was strong agreement among the informants regarding ethnomedicinal uses of plants, with Factor of Informant Consensus (FIC) values ranging from 0.50 to 0.66, with the highest number of species (49) being used for the treatment of digestive system disorders (FIC 0.66). In contrast, the least agreement (FIC = 0.50) between informants regarding therapeutic uses was observed for plants used to treat urinary disorders. The present study was compared with 43 prior ethnomedicinal studies, conducted both nationally and in neighboring countries, and the results revealed that the Jaccard index (JI) ranged from 1.65 to 33.00. The highest degree of similarity (33.00) was found with another study conducted in Bangladesh, while the lowest degree of similarity (1.65) was found with a study conducted in Pakistan. This study recorded 12 new ethnomedicinal plant species, of which 6 have never been studied pharmacologically to date.

**Conclusions:**

This study showed that the Pangkhua community still depends substantially on ethnomedicinal plants for the treatment of various ailments and diseases and that several of these plants are used in novel ways or represented their first instances of use for medicinal applications.

**Electronic supplementary material:**

The online version of this article (10.1186/s13002-019-0287-2) contains supplementary material, which is available to authorized users.

## Background

Traditional herbal medicine in Bangladesh has strong cultural and religious foundations. It manifests in different ways among indigenous groups in their ritual or ceremonial practices, spiritual practices, and self-healing practices. Indigenous communities have utilized this local knowledge for centuries to cure different diseases. Reportedly, more than 80% of the Bangladeshi use non-allopathic medicines (Ayurveda, Siddha, Unani, and homeopathy) for their healthcare, with herbs constituting a major ingredient of these alternative systems of medicine [1]. Bangladesh is a country that is considered rich in medicinal plant genetic resources, by virtue of its favorable agroclimatic conditions and seasonal diversity. With productive soils and a tropical climate, more than 5000 angiospermic plant species have been recorded in the country [2], of which about 250 have documented use in traditional medicine systems [3]. About 75% of the country’s total population lives in rural areas, and almost 80% is dependent on natural resources (e.g., medicinal plants) for their primary healthcare needs [4]. Rural/indigenous peoples are capable of identifying many species of plants yielding various products, including food, firewood, medicine, forage, and tools for daily needs. With such a high demand for herbal medicines, the medicinal plant sector has been cited as the most promising business sector in Bangladesh [5], with more than 500 companies producing herbal medicines, yet despite the biodiversity described above, more than 90% of the plants and products needed to meet domestic demands are imported from other countries, such as India, Nepal, and Pakistan.

Many indigenous Bangladeshi live in deep forest zones. They include those people living within the three Chittagong Hill Tract districts (CHTs) of south-eastern Bangladesh, within which there are 12 indigenous communities [6]. The smallest of these communities is the Pangkhua, who dwell in the remote Pangkhua paras, an isolated part of the Bilaichari Upazilla of the Rangamati CHT. In the wet season, the only way to reach Pangkhua paras is by motorboat, taking 6 h, while in the dry season it takes more than 8 h on foot. Like other remote communities, the Pangkhua have their own distinct traditional healthcare system and practices. In fact, the nearest conventional medicine facility is in Belaichari Upazilla sadar, the only Government health facility nearby (about 15 km), with basic health facilities. Services there are provided by two medical practitioners alongside three paramedics. The Pangkhua people thus have inadequate access to modern treatments, and in any case, allopathic medicine is largely unaffordable to them. Traditional medicinal knowledge, on the other hand, is orally transmitted from one generation to the next. Typically, every elderly man and woman of the community can prepare herbal formulations for the treatment of common ailments, such as fever, cough, cold, dysentery, diarrhea, and gastritis. Typically, they visit professional healers only when they suffer from more serious symptoms or conditions, such as jaundice, cholera, malaria, or cancers. The headmen (*karbari*) of each village also act as professional healers. In fact, many Pangkhua believe that they lose their community spirit if they receive allopathic care. Local government has had to enforce modern treatment in instances of contagious disease.

Several studies on ethnomedicinal plants of Bangladesh have been conducted in the past, and comprehensive works have already been published [7–13]. However, few studies focus on the Rangamati district [10, 14, 15] with almost nothing on the Pangkhua indigenous community. With this in mind, the Pangkhua indigenous community was selected for the present study, as their ethnomedicinal practices have not been thoroughly investigated to date. It was important to ascertain who among them represent the custodians of such knowledge and to document their uses of medicinal plants. To the best of our knowledge, this is the pioneer quantitative documentation of medicinal plants in the studied area.

## Methods

### Study area

**Fig. 1:**
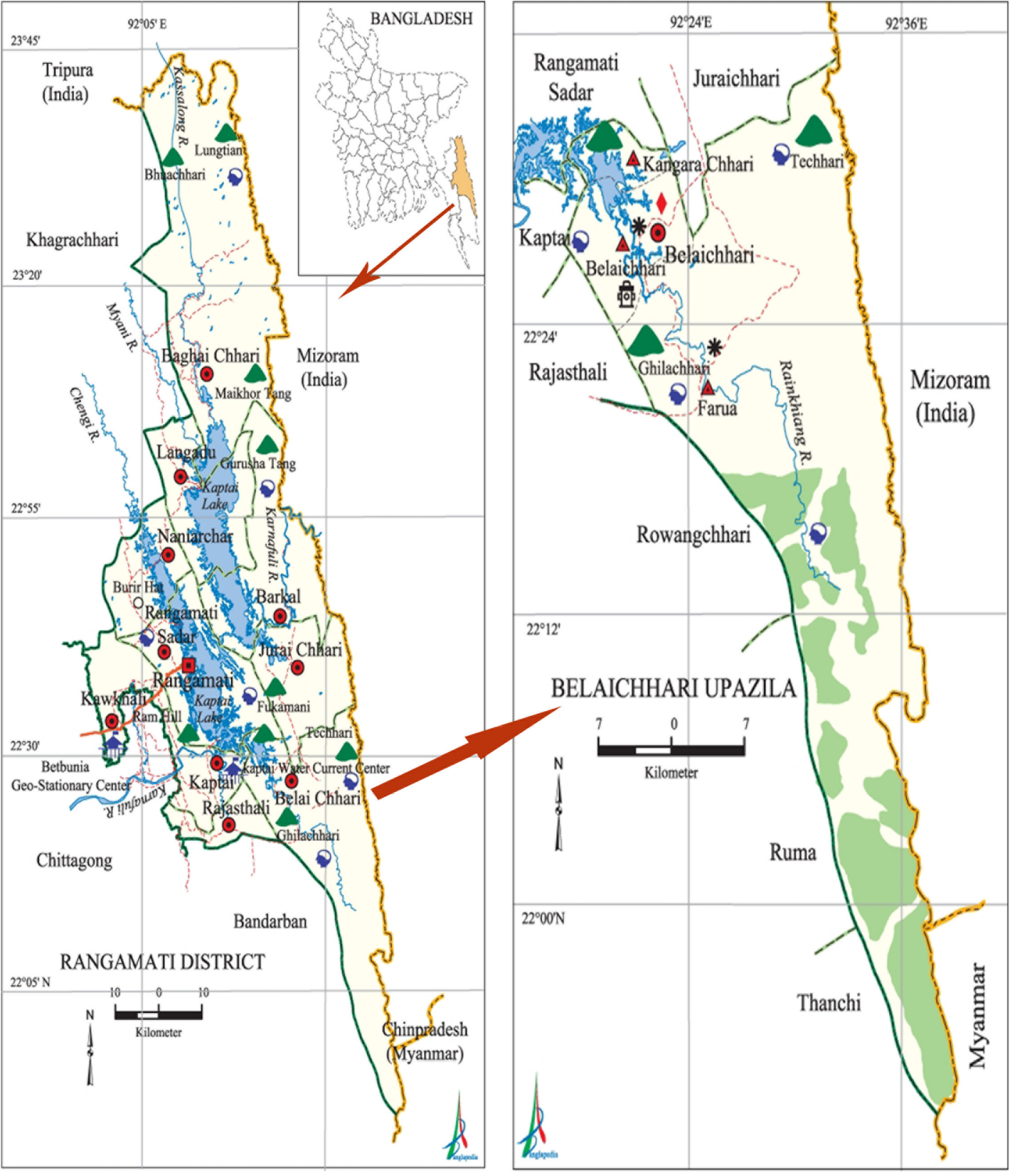
A map of the studied area [16]

The present study was carried out in the Pangkhua areas of the Belaichhari Upazila within the Rangamati District (Fig. 1). This district is part of the Chittagong division and Chittagong Hill Tracts. Belaichhari thana (now an upazila) was established in 1976. It consists of 3 Union parishads, 9 mouzas and 30 villages. The Belaichhari Upazila is situated approximately between 20° 50′ and 22° 35′ N latitude and between 90° 38′ and 92° 17′ E longitude. The Rainkhiang is the main river of the upazilla. The district lies in the south-east of Bangladesh and has a tropical monsoon climate. There are three main seasons: the dry season (November to March), which is sunny and dry; the pre-monsoon (April to May), which is very hot and sunny with occasional showers; and the rainy season (June to October), which is warm, cloudy, and wet. Temperatures of the Belaichhari Upazila are moderate, with a mean monthly average temperature in Rangamati of 25.8 °C and annual monthly average temperatures ranging from 13.4 to 34.6 °C. The mean annual rainfall is 2865.4 mm, with mean monthly maxima and minima of 679 mm (July) and 7.4 mm (January), respectively [16].

### Methods of study

The success of ethnobotanical documentation depends on the cooperative relationship between the researcher and local informant. Knowledgeable informants are very important for the study of ethnobotany [17, 18]. Various techniques are recommended for ethnobotanical studies, including (i) direct or participant observation, (ii) checklist interview, (iii) group interview, (iv) field interview, (v) plant interview, and (vi) market survey [19, 20]. All of these techniques were followed in this study except the use of checklist interviews. The interview is a dynamic process involving spoken interactions between two or more people. In general, open-ended and semi-structured techniques were followed. Initial contacts are very important to understand an area and its people. Initial contacts were made with headmen, teachers, and students within the area to select informants. Upon identification of informants, if necessary, interpreters were also appointed. Ethnobotanical information regarding the usage of medicinal plants available in the local area for treating various ailments and diseases was collected through direct interviewing of traditional healers and other informants possessing traditional knowledge about medicinal plants. During the interviews, information was noted using data documentation sheets; in addition, audio recording was performed with a digital voice recorder. Contact in the field was conducted over a total of 43 days, in different seasons, with interviews conducted in the Chittagonian language, accompanied by a local student (Bathue Pankhua) and with consultancy with a local doctor (Dr. Mizanur Rahman).

### Quantitative analysis

To analyze the data, we adopted the following quantitative ethnobotanical techniques:

### Factor of informant consensus (FIC)

The level of homogeneity between information provided by different informants was calculated using the factor of informant consensus (FIC) [21, 22]. It is calculated as FIC = Nur – Nt/(Nur – 1), where Nur is the number of use reports from informants for a particular plant-use category and Nt is the number of taxa or species associated with that plant-use category across all informants. FIC values range between 0 and 1, with FIC = 1 indicating the highest level of informant consensus. A high value (close to 1) indicates that relatively few taxa (or, more usually, species) are used by a large proportion of informants, while a low value indicates that informants differ on the taxa to be used in treatment within a category of illness. Therefore, if informants use few taxa, then a high degree of consensus is reached and medicinal tradition is thus viewed as well-defined [23].

### Jaccard index (JI)

We also wished to calculate similarities between our studies with prior ethnobotanical studies carried out in other parts of Bangladesh, as well as those from neighboring countries. This may be expressed using the Jaccard index (JI), which uses the following formula [24, 25]:

JI = C × 100/A + B − C, where, *A* is the recorded number of species of the current study area *a*, *B* is the documented number of species of another study area *b*, and *C* is the number of species common to both areas *a* and *b*.

## Results

### Enumeration of taxa

The ethnobotanical survey was carried out three times during summer and winter seasons from January 2016 to September 2017. All plant materials were collected and identified through expert consultation, by comparison with herbarium specimens and through use of literature references. Following preservation, plant materials were numbered and deposited as voucher specimens in the Chittagong University Herbarium. Descriptions and current nomenclature were compared with the recent “Dictionary of Plant Names of Bangladesh-Vascular Plants” [2] and with www.theplantlist.org. The ethnomedicinal value of each plant was cataloged as follows: botanical name (with voucher number in brackets), Bangla name, Pangkhua name, family, habit, plant part(s) used, disease(s)/illness treated, usage information, and prior documentation in the allied literature (Table 1).

**Table 1:** List of the ethnomedicinal plant species used by the Pangkhua community of the Rangamati district, Bangladesh

Scientific name, voucher number, family and conservation status	Bangla name	Pangkhua name	Habit	Parts used	Number of citations per ailment category	Usage information	FC	UR	Allied literature
*Acorus calamus* L. (bot-t1015), Acoraceae, least concern	Bach	Thit	H	Rz	Anthelmintic (5), gastritis (7)	A decoction of the rhizome is taken at a dose of one cupful daily for 7 days to treat gastritis, and as an anthelmintic.	12	2	1▲2●3▲4▲5●6▲7♣8▲9▲10▲10●11●12♣13●14●15●16▲17●18▲19●20●21●22▲23●24●25●26▲27●28●29●30●31●32●33●34●135▲36●37●38●39●40●41●42●43●
*Aegle marmelos* (L.) Correa (bot-t1044), Rutaceae	Bel	Highshu	T	Fr	Digestive (3), asthma (1)	Juice of the fruit is taken (as much as possible) for 7 days as a digestive. A decoction of green fruits along with the bark of *Terminalia arjuna* is taken at a dose of one teaspoonful twice daily to treat asthma.	4	2	1▲2●3●4●5♣6▲7▲8▲9●10♣11▲12●13●14▲15♣16●17●18●19▲20▲21●22♣23●24●25●26●27●28●29♣30●31●132●33♣34●35●36●37♣38●39●40▲41▲42♣43●
*Albizia myriophylla* Benth (bot-t10108), Fabaceae	Titulya koroi	Unknown	*T*	L	Asthma (8), leucorrhoea (4)	Leaf juice, along with honey and other (unknown) ingredients is ktaken at a dose of one cupful daily to treat asthma. A paste of the leaves is smeared around the vaginal area to treat leucorrhoea.	12	2	1●2●3●4●5●6●7●8●9●10●11●12●13●14●15●16●17●18●19●20●21●22●23●24●25●26●27●28●29●30●31●32●33●34●35●36●37●38●39●40●41●42●43●
*Aloe vera* (L.) Burm.f. (bot-t1032), Xanthorrhoeaceae	Ghritkumari	Cladora	H	L	Burns (12)	Juice of the leaves is applied to burns.	12	1	1●2●3♣4♣5●6●7▲8●9●1011●12●13●14●15▲16●17●18●19●20●21●22●23●24●25●26●27●2829●30●31●32●33●34●35●36●37●38●39●40●41●42●43●
*Alpinia conchigera* Griff. (bot-t1085), Zingiberaceae		Aidul	H	Rz	Diarrhea (5), dysentery (6)	A decoction of the rhizome is taken orally for 5 to 7 days at a dose of one cupful three times daily for the treatment of diarrhea. The same dose is given for 3 to 4 days in dysentery.	11	2	1●2●3●4●5●6●7●8●9●10▲11●12●13●14●15●16●17●18●19●20●21●22▲23●24●25●26●27●28●29●30●31●32●33●34●35●36●37●38●39●40●41●42●43●
*Alpinia nigra* (Gaertn.) Burtt (bot-t1084), Zingiberaceae	Jangliada	Bawnkawr	H	Rz	Cough and colds (10)	A decoction of the rhizome with honey is taken at a dose of one teaspoonful three times daily for 3 days.	10	1	1●2●3●4●5●6●7●8●9●10●11●12●13●14●15●16●17●18●19●20●21●22●23●24●25●26●27●28●29●30●31●32●33●34●35●36●37●38●39●40●41●42●43●
*Alternanthera pungens* Kunth (bot-t10109), Amaranthaceae	Kakishak	Unknown	*H*	WP	Kidney problems (8)	A decoction of the plant is taken for kidney problems.	8	1	1●2●3●4●5●6●7●8●9●10●11●12●13●14●15●16●17●18●19●20●21●22●23●24●25●26●27●28●29●30●31●32●33●34●35●36●37●38●39●40●41●42●43●
*Alysicarpus monilifer* (L.) DC. (bot-t10101), Fabaceae	Alisimon	Unknown	H	WP	Fever (4), jaundice (3), diabetes (4)	Boiled plants are taken directly: as much as possible is consumed to treat fever; the juice of the plant along with other (unknown) ingredients is given for the treatment of jaundice. A decoction of the plant is taken to treat diabetes.	11	3	1●2♣3●4●5●6●7●8●9●10●11●12●13●14●15●16●17●18●19●20●21●22●23●24●25●26●27●28●29●30●31●32●33●34●35●36●37●38●39●40●41●42●43●
*Amaranthus spinosus* L. (bot-t1049), Amaranthaceae	Kantanotey	Unknown	H	L	Eczema (26), dysuria (6)	Leaf juice along with sugar or molasses is applied to eczema. A decoction of leaves is taken in dysuria.	32	2	1●2●3●4▲5♣6♣7▲8▲9●1011●12**▲**13●14**▲**15▲16▲17●18●19●20●21●22▲23●24●25●26▲27▲28●▲29●30●31●32▲33●34●35●36●37●38●39●40●41●42●43●
*Amberboa moschata*(L.) DC (bot-t10104), Asteraceae	Jam	Unknown	H	R	Cancer (1), menstrual problems (3), ulcers (2)	The root is directly eaten for menstrual problems. A decoction of the root is given at a dose of one cupful twice daily for the treatment of cancer and ulcers.	6	3	1●2●3●4●5●6●7●8●9●10●11●12●13●14●15●16●17●18●19●20●21●22●23●24●25●26●27●28●29●30●31●32●33●34●35●36●37●38●39●40●41●42●43●
*Ammannia multiflora* Roxb (bot-t10110), Lythraceae, least concern	Niamul	Unknown	*H*	WP	Burns (3), backache (1)	A paste of the plant is applied to burns, and the same formulation, along with mustard oil, is applied to treat backache.	4	2	1●2●3●4●5●6●7●8●9●10●11●12●13●14●15●16●17●18●19●20●21●22●23●24●25●26●27●28●29●30●31●32●33●34●35●36●37●38●39●40●41●42●43●
*Amorphophallus paeoniifolius* (Dennst.) Nicolson. (bot-t1086), Araceae, least concern	Olkachu	Ranilawngbal	H	Tb	Diarrhea (20)	The juice extracted from the tuber is given for the treatment of diarrhea.	20	1	1●2●3●4●5●6●7●8●9●10●11●12●13●14●15●16●17●18●19●20●21●22●23●24●25●26●27●28●29●30●31●32●33●34●35●36●37●38●39●40●41●42●43●
*Anacardium occidentale* L. (bot-t1087), Anacardiaceae	Kajubakam	Guestunut	T	B	Dysentery (13), diarrhea (9)	A bark decoction is taken in cases of dysentery and diarrhea.	22	2	1●2●3●4●5●6●7●8●9●10●11●12●13●14●15●16●17●18●19●20●21●22●23●24●25●26●27●28●29●30●31●32●33●34●35●36●37●38●39●40●41●42●43●
*Ananas comosus* (L.) Merr. (bot-t1016), Bromeliaceae	Anaras	Lathy	H	L, Fr	Anthelmintic (14)	One teaspoonful of juice from the leaves and fruit is taken once daily for 3 days as an anthelmintic.	14	1	1●2●3●4▲5●6●7▲8●9▲10▲11●12●13●14●15♣16●17●18●19▲20●21●22♣23●24●25●26♣27●28●29●30●31●32●33●34●35●36●37●38▲39●40▲41●42●43●
*Antidesma velutinosum* Blume (bot-t10111), Phyllanthaceae	Pashmi salishiabuka	Nandul	T	Fr	Menstrual disorders (4), fever (2)	A decoction of the fruit (about 3 teaspoonfuls three times daily for 7 days) is prescribed to cure menstrual problems and high fever.	6	2	1●2●3●4●5●6●7●8●9●10●11●12●13●14●15●16●17●18●19●20●21●22●23●24●25●26●27●28●29●30●31●32●33●34●35●36●37●38●39●40●41●42●43●
*Areca catechu* L. (bot-t1035), Arecaceae	Supari	Panthong	T	Fr	Carminative (12)	Fruits are taken as a carminative.	12	1	1▲2●3●4▲5●6●7▲8▲9●10●11●12●13●14●15●16●17●18●19●20●21●22●23●24●25●26●27●28●29●30●31●32●33●34●35●36●37●38●39●40●41●42●43●
*Argyreia splendens* (Hornem.) Sweet (bot-t10112), Convolvulaceae	Chottorupatola	Ramsingkholong	C	L	Ulcers (6)	One cupful of the leaf decoction is taken twice daily before meals for 15 days to treat ulcers.	6	1	1●2●3●4●5●6●7●8●9●10●11●12●13●14●15●16●17●18●19●20●21●22●23●24●25●26●27●28●29●30●31●32●33●34●35●36●37●38●39●40●41●42●43●
*Artocarpus heterophyllus* Lam. (bot-t1083), Moraceae	Kathal	Luwe	T	L	Skin disease (16)	A paste of the young leaves is applied to the affected areas of skin.	16	1	1●2●3●4●5●6●7▲8●9●10●11●12●13●14●15●16●17●18●19●20●21●22●23●24●25●26●27●28●29●30●31●32●33●34●35●36●37●38●39●40●41●42●43●
*Azadirachta indica* A. Juss. (bot-t1079), Meliaceae	Neem	Neem	T	L, B	Scabies (11), malaria (8)	Boiled leaves and bark are used in a bath for the treatment of scabies. A decoction of the leaves is taken in malaria.	19	2	1●2▲3♣4♣5♣6▲7♣8●9♣10♣11♣12♣13♣14♣15♣16▲17▲18●19●20♣21♣22●23●24●25●26♣27●28●29▲30●31●32●33▲34●35●36●37●38♣39●40●41●42♣43●
*Azolla pinnata* R. Br. (bot-t1097), Salviniaceae, least concern	Jalpai	Anuran	Herb	WP	Skin disease (3), diarrhea (6), pneumonia (3)	A paste of the plant is rubbed on the affected area in skin diseases. A decoction of the plant is taken with honey to treat diarrhea and pneumonia.	12	3	1●2●3●4●5●6●7●8●9●10●11●12●13●14●15●16●17●18●19●20●21●22●23●24●25●26●27●28●29●30●31●32●33●34●35●36●37●38●39●40●41●42●43●
*Baliospermum solanifolium* (Burm.) Suresh (bot-t1080), Euphorbiaceae	Danti	Ankhu	US	L	Scabies (20)	Boiled leaves are used in a bath for the treatment of scabies.	20	1	1●2●3●4●5●6●7●8●9●10●11●12●13●14●15●16●17●18●19●20●21●22●23●24●25●26●27●28●29●30●31●32●33●34●35●36●37●38●39●40●41●42●43●
*Bauhinia acuminata* L. (bot-t1033), Caesalpiniaceae	Kanchan	Senpui	T	R	Burns (22)	A decoction of the root is boiled with coconut oil and applied to burns.	22	1	1●2●3●4●5●6●7●8●9●10●11●12●13●14●15●16●17●18●19●20●21●22●23●24●25●26●27●28●29●30●31●32●33●34●35●36●37●38●39●40●41●42●43●
*Bauhinia scandens* L. (bot-t10113), Fabaceae	Gundagilla	Dimonong	T	R, L	Respiratory problems (4), rheumatic pain (5)	A decoction of the preparedroot is given to treat respiratory problems. The juice of the leaves is taken at a dose of one teaspoonful daily for 7 days for relief of rheumatic pain.	9	2	1●2●3●4●5●6●7●8●9●10●11●12●13●14●15●16●17●18●19●20●21●22●23●24●25●26●27●28●29●30●31●32●33●34●35●36●37●38●39●40●41●42●43●
*Bidens pilosa* L. (bot-t10114), Asteraceae	Bidenlosa	Sakbal	H	WP	Cough and colds (11)	A decoction prepared from the plant is taken to treat coughs and colds.	11	1	1●2●3●4●5●6●7●8●9●10●11●12●13●14●15●16●17●18●19●20●21●22●23●24●25●26●27●28●29●30●31●32●33●34●35●36●37▲38●39●40●41●42●43●
*Biophytum sensitivum* (L.) DC. (bot-t1096), Oxalidaceae	Bannaringa	Arikbel	H	L	Wounds and cuts (24)	A paste of the leaves is applied to wounds and cuts to stop bleeding.	24	2	1●2●3●4●5●6●7●8●9●10●11●12●13●14●15●16●17●18●19●20●21●22●23●24●25●26●27●28●29●30●31●32●33●34●35●36●37●38●39●40●41●42●43●
*Blumea balsamifera* (L.) DC. (bot-t1052), Asteraceae	Nagor chandal	Pangkhaper	H	L	Conjunctivitis (29)	two to three drops of fresh leaf juice is dropped into the eyes in chronic eye disease.	29	1	1●2●3●4●5●6●7●8●9●10▲11●12●13●14●15●16●17●18●19●20●21●22●23●24●25●26●27●28●29●30●31●32●33●34●35●36●37●38▲39●40●41●42●43●
*Blumea lacera* (Burm.f.) DC. (bot-t1017), Asteraceae	Kukursunga	Vaiankasa	H	L	Anthelmintic (9), cough (3)	Two teaspoonfuls of leaf juice are taken three times a day for 10 days as an anthelmintic and to treat cough.	12	2	1●2●3▲4●5●6▲7●8●9●10▲11**▲**12●13●14●15●16●17●18●19●20**▲**21●22●23●24●25●26●27●28●29●30●31●32●33●34●35●36●37●38●39●40●41●42●43●
*Bombax ceiba* L. (bot-t1058), Bombacaceae	Shimul	Ompang	T	R	Gonorrhea (12)	Two to three teaspoonfuls of root juice are taken twice a day to treat gonorrhea.	12	1	1●2●3●4▲5●6●7●8●9●10●11▲12♣13●14●15▲16●17●18●19●20**▲**21●22●23●24●25●26●27●28●29●30●31●32●33●34●35●36**▲**37●38▲39●40▲41●42**▲**43●
*Breonia chinensis* (Lam.) Capuron (bot-t1088), Rubiaceae, least concern	Kadam	Zacibgoar	T	B	Diarrhea (7)	A decoction of bark with honey and salt is taken orally at a dose of one cupful twice daily for 7 days.	7	1	1●2●3●4●5●6●7●8●9●10●11●12●13●14●15●16●17●18●19●20●21●22●23●24●25●26●27●28●29●30●31●32●33●34●35●36●37●38●39●40●41●42●43●
*Buddleja asiatica* Lour. (bot-t1084), Buddlejaceae	Budbhota	Langtel	US	R	Skin disease (6), pneumonia (5)	An infusion of the root is applied in skin disease. A decoction of the root is taken to treat pneumonia.	11	2	1●2●3●4●5●6●7●8●9●10▲11▲12●13●14●15●16●17●18●19●20●21●22▲23●24●25●26●27●28●29●30●31●32●33●34●35●36●37●38●39●40●41●42●43●
*Butea monosperma* (Lam.) Taub. (bot-t1025), Fabaceae	Palas	Tuangtoapar	T	B, Sd	Anthelmintic (4), dysentery (10), urinary infections (4), cough (3)	Juices prepared from bark and seed are used as an anthelmintic and to treat dysentery. In addition, a decoction of the bark is taken to treat urinary infections and cough.	21	4	1▲2●3●4●5●6▲7▲8●9▲10●11●12●13●14●15●16●17●18●19●20♣21●22▲23●24●25●26●27♣28●29▲30●31●32●33♣34●35●36●37●38●39●40●41●42●43●
*Cajanus cajan* (L.) Millsp. (bot-t1055), Fabaceae	Arhar	Koklang	S	L, Sd	Gastritis (23), jaundice (11)	One cupful of leaf extract is taken twice a day for 5 days before meals in gastritis. Cooked seeds are taken directly to treat jaundice.	34	2	1●2●3●4▲5●6●7♣8●9▲10●11●12●13●14●15●16●17♣18●19●20♣21●22♣23●24●25●26●27●28●29●30●31●32●33●34●35●36●37●38●39●40●41●42**▲**43●
*Callicarpa tomentosa* (L.) L. (bot-t1094), Lamiaceae	Makanchi	Lankia	T	St	Diarrhea (14)	A decoction of the stem is taken at a dose of one cupful twice daily for 7 days.	14	1	1●2●3●4●5●6●7●8●9●10●11●12●13●14●15●16●17●18●19●20●21●22●23●24●25●26●27●28●29●30●31●32●33●34●35●36●37●38●39●40●41●42●43●
*Calotropis gigantea* (L.) Dryand. Ait.f. (bot-t1069), Asclepiadaceae	Akanda	Napal	S	L	Rheumatic pain (24)	Leaves are applied to the affected area twice a day for 3 days for the treatment of rheumatic pain.	24	1	1♣2●3●4♣5●6♣7▲8▲9▲10♣11●12●13♣14▲15●16●17●18●19●20♣21●22♣23●24♣25●26♣27●28●29●30●31●32●33●34●35●36●37●38●39●40●41●42**▲**43●
*Calotropis procera* (Aiton) Dryand. (bot-t1026), Asclepiadaceae	Akanda	Napal	S	F, L	Asthma (9), snake bite (5)	Flower extracts are used in asthma. A decoction of leaves is used to treat snake bite.	14	2	1●2●3●4●5●6●7●8●9●10▲11●12●13●14●15▲16●17●18●19●20●21●22▲23●24●25●26●27●28●29●30●31●32●33●34●35♣36●37●38●39●40●41●42●43●
*Canna indica* L. (bot-t1018), Cannaceae	Kataboti	Bawnkawr	H	Rz	Anthelmintic (10), asthma (12)	Two teaspoonfuls of the rhizome extract are taken once daily for 5 days early in the morning before meals, as an anthelmintic and to treat asthma.	22	2	1●2●3●4●5●6●7●8●9●10●11●12●13●14●15●16●17●18●19●20●21●22●23●24●25●26●27●28●29●30●31●32●33●34●35●36●37●38●39●40●41●42●43●
*Carica papaya* L. (bot-t1045), Caricaceae	Pepe	Colra	H	Fr	Digestive and dysentery (11)	Ripe fruits are eaten directly as a digestive. Boiled green fruits are taken with leaves of *Centella asiatica* to treat dysentery.	11	2	1▲2●3●4♣5♣6●7▲8●9♣10●11●12●13●14●15●16●17●18●1219●20●21●22●23●24●25●26▲27●28●29●30●31●32●33●34●35●36●37●38●39●40●41●42●43●
*Cassia fistula* L. (bot-t1065), Caesalpiniaceae	Sonalu	Enkhang	T	Fr	Jaundice (8), dysentery (12)	An extract of the fruit is taken at a dose of one cupful three times daily to treat jaundiced patients. The bark juice is used for the treatment of dysentery in cattle.	20	2	1●2●3●4▲5▲6●7♣8▲9●10♣11●12**▲**13●14●15●16♣17●18♣19▲20♣21●22●23●24▲25●26●27●28♣29▲30●31●32●33▲34●35♣36●37●38▲39●40●41▲42♣43●
*Centella asiatica* (L.) Urb. (bot-t1053), Apiaceae, least concern	Thunkuni	Changchi khiat	H	WP	Conjunctivitis (4), dysentery (10), impotence (4), asthma (4)	2–3 drops of plant juice are dropped twice daily into the eyes to treat conjunctivitis. The whole plant is eaten (as much as possible) as a vegetable to treat dysentery and impotence. A decoction of the whole plant, along with honey, is taken to treat asthma.	22	4	1♣2▲3●4♣5♣6♣7♣8♣9▲10♣11▲12♣13●14♣15▲16●17♣18●19●20♣21●22♣23●24●25●26♣27▲28♣29●30●31♣32▲33▲34●35●36♣37▲38●39●40♣41●42▲43♣
*Cheilocostus speciosus* (J.Koenig) C.D.Specht (bot-t1048), Costaceae	Keu	Terpimungkhorol	H	L, St	Earache (8)	The juice of leaves and stems is taken at a dose of one teaspoonful three times daily for 3 days to treat ear pain.	8	1	1●2●3▲4●5●6●7●8●9●10▲11●12●13●14●15●16●17●18●19●20●21●22●23●24●25●26●27●28●29●30●31●32●33●34●35●36●37●38●39●40●41●42●43●
*Cinnamomum tamala* (Buch.-Ham.) T.Nees & Eberm. (bot-t1095), Lauraceae	Tejpata	Matuinana	T	L	Cough and cold (12)	A leaf decoction is taken orally to treat coughs and colds.	12	2	1●2●3●4●5▲6▲**7**●8●9▲10●11●12●13●14●15●16●17●18●19●20●21●22●23●24●25●26●27●28●29●30●31●32●33●34●35●36●37●38●39●40●41●42●43●
*Cissus repens* Lam. (bot-t1046), Vitaceae	Marmaria pata	Puipal	C	R	Dog bites (12)	A paste of the root is applied to dog bites.	12	1	1●2●3●4●5●6●7●8●9●10●11●12●13●14●15●16●17●18●19●20●21●22●23●24●25●26●27●28●29●30●31●32●33●34●35●36●37●38●39●40●41●42●43●
*Citrus aurantifolia* (Christm). Swingle (bot-t1059), Rutaceae	Lebu	Charmum	S	Fr, L	Headache (9), Malaria (3)	The fruit juice is taken to treat malaria. The leaf paste is rubbed to the forehead in patients with headache.	14	2	1●2●3●4▲5●6●7●8●9●10●11●12●13●14●15●16●17●18●19●20▲21●22●23●24●25●26▲27●28●29●30●31●32●33●34●35●36●37●38●39●40●41●42●43●
*Citrus maxima* (Burm.) Merr. (bot-t1090), Rutaceae	Jambura	Sherthur	T	Fr	Fever (17), cough (5)	For treatment of fever and cough, fruits are directly eaten with a trace amount of salt and red chili.	22	2	1●2●3●4▲5●6●7●8●9●10●11●11●12●13●14●15●16●17●18●19●20●21●22●23●24●25●26●27●28●29●30●31●32●33●34●35●36●37●38●39●40●41●42●43●
*Clerodendrum viscosum* Vent. (bot-t1024), Verbenaceae	Bhat	Kuidim	S	L	Anthelmintic (8), cough (7), dysentery (7)	A leaf extract is taken as an anthelmintic; the leaf juice is taken at a dose of two teaspoons three times daily for 7 days to treat cough and dysentery.	22	3	1●2●3●4♣5♣6▲7▲8●9●10●11●12♣13●14▲15▲16●17▲18●19▲20●21▲22♣23●24●25●26▲27●28♣29●30●31●32●33●34●35●36▲37●38●39●40●41♣42♣43▲
*Clerodendrum indicum* (L.) Kuntze (bot-t1096), Lamiaceae	Bamunhatti	Senkuidem	S	L	Cough and asthma (13)	A decoction prepared from the leaves is given to treat cough and asthma.	13	2	1●2●3♣4♣5●6▲7●8●9●10▲11●12●13●14●15●16●17●18●19●20●21●22●23●24●25●26●27●28●29●30●31●32●33●34●35●36●37●38●39●40●41●42●43●
*Coccinia grandis* (L.) Voigt (bot-t1070), Cucurbitaceae	Telakucha	Thiback	C	L	Joint pain (33)	Baked leaves are applied in joint pain.	33	1	1●2●3●4▲5●6●7▲8●9●10●11●12●13●14♣15▲16●17●18●19▲20▲21●22●23●24●25▲26▲27●28▲29●30●31●32●33●34●35●36●37●38●39●40●41●42▲43●
*Cocos nucifera* L. (bot-t1037), Arecaceae	Narkel	Lukluk	H	Fr	Carminative (8), digestive (6), fever (2)	Green coconut water is taken as a carminative and digestive, and is also taken during fever.	16	3	1●2●3●4♣5▲6●7▲8●9●10●11▲12**▲**13●14▲15▲16●17●18●19●20●21●22●23●24●25●26●27●28●29●30●31●32●33●34●35●36●37●38●39●40●41●42●43●
*Commelina diffusa* Burm.f. (bot-t1029), Commelinaceae, least concern	Monayna Kanshira	Dongjal	H	St	Boil in the ear canal (4)	An extract prepared from tender stems is applied to the ear for the treatment of boils.	4	1	1●2●3●4●5●6●7●8●9●10●11●12●13●14●15●16●17●18●19●20●21●22●23●24●25●26●27●28●29●30●31●32●33●34●35●36●37●38●39●40●41●42●43●
*Coriandrum sativum* L. (bot-t1071), Apiaceae	Dhaniya	Changroi	H	Fr	Stomachache (32)	Dry fruits are eaten with betel leaf to obtain relief from stomachache.	32	1	1▲2●3●4●5●6●7♣8●9●10▲11▲12●13●14▲15●16●17●18●19●20●21●22●23●24●25●26●27●28●29●30●31●32●33●34●35●36●37●38●39●40●41●42●43●
*Crassocephalum crepidioides* (Benth.) S. Moore (bot-t1090), Asteraceae	Duubbecrepi	Baiunkasa	H	WP	Stomach pain (33)	The plant extract is taken as a remedy for stomach pain.	33	1	1●2●3●4●5●6●7●8●9●10●11●12●13●14●15●16●17●18●19●20●21●22●23●24●25●26●27●28●29●30●31●32●33●34●35●36●37●38●39●40●41●42●43●
*Crateva unilocularis*Buch.-Ham (bot-t10115), Capparaceae	Ekkosha barun	Ushsak	*T*	L	Urinary disorders (4), high blood pressure (2)	An extract of the leaves is taken three times a day for a week for the treatment of urinary problems and high blood pressure.	6	2	1●2●3●4●5●6●7●8●9●10●11●12●13●14●15●16●17●18●19●20●21●22●23●24●25●26●27●28●29●30●31♣32●33●34●35●36●37●38●39●40●41●42●43●.
*Crotalaria pallida* Aiton (bot-t1060), Fabaceae	Jhanjuni	Rockac pabel	H	R	Indigestion (13)	A root extract is taken at a dose of one cupful daily for 15 days to treat indigestion.	13	1	1●2●3●4●5●6●7●8●9●10●11▲12●13●14●15●16●17●18●19●20●21●22●23●24●25●26●27●28●29●30●31●32●33●34●35●36●37●38●39●40●41▲42●43●
*Cucurbita maxima* Duchesne (bot-t1034), Cucurbitaceae	Mistikumra	Mypore	Herb	L, F, Fr, Sd	Burns and boils (22)	The fruit pulp is useful in burns and boils. The young leaves, flowers and fruits are cooked as vegetables. Fried seeds are eaten. Fruits are boiled to make smashed (bharta). The fruit skin is also cooked as a vegetable.	22	1	1●2●3●4●5●6●7●8●9●10●11●12●13●14●15●16●17●18●19●20●21●22●23●24●25♣26●27●28●29●30●31●32●33●34●35●36●37●38●39●40●41●42●43●
*Curcuma caesia* Roxb. (bot-t1092), Zingiberaceae	Kalahalood	Aailiedum	H	Rz	Fever (5), Tumor (1), snake bite (1)	A rhizome decoction is used orally, at a dose of one teaspoonful twice daily with cow’s milk to treat fever. A paste of the rhizome is used to treat tumor and snake bite.	7	1	1●2●3●4●5●6●7●8●9●10●11●12●13●14●15●16●17●18●19●20●21●22●23●24●25●26●27●28●29●30●31●32●33●34●35●36●37●38●39●40●41●42●43●
*Curcuma longa* L. (bot-t1050), Zingiberaceae	Halud	Chang	H	Rz, F	Eczema (11), dysentery (12), coughs, cold and fever (19), laxative (3)	The rhizome is cooked and taken to treat dysentery. Flowers are used as additives in curries. A paste of the rhizome is used for the treatment of eczema; juice of the rhizome is taken (one teaspoon three times a day for 7 days) to cure cough, colds and fever and is also taken as a laxative.	45	6	1●2●3●4♣5▲6●7♣8♣9▲10▲11**▲**12**▲**13▲14●15♣16●17●18●19●20▲21●22**▲**23●24●25●26▲27●28●29●30●31●32●33▲34●35●36●37●38▲39●40▲41●42♣43▲
*Curcuma zedoaria* (Christm.) Roscoe (bot-t1091), Zingiberaceae	Sothi	Aaiangpor	H	Rz, F	Diarrhea (8), coughs (2)	A decoction of the rhizome is given in diarrhea. Flowers are directly eaten with rice to treat coughs.	10	2	1●2●3●4●5●6●7●8●9●10▲11●12●13●14●15●16●17●18●19●20●21●22**▲**23●24●25●26●27●28●29●30●31●32●33●34●35●36●37●38●39●40●41●42●43●
*Cyperus rotundus* L. (bot-t1089), Cyperaceae, least concern	Mutha	Belring	H	WP	Diarrhea and dysentery (20)	A decoction prepared from the whole plant mixed with rice-washed water is taken at a dose of one cupful three times daily until cure, in cases of diarrhea and dysentery.	20	2	1▲2●3●4♣5●6●7▲8●9●10▲11●12●13●14●15●16●17●18●19●20●21●22●23●24●25●26●27●28●29●30▲31●32●33●34▲35●36▲37●38●39●40●41●42▲43●
*Derris indica* (Lamk.) Bennet. (bot-t1031), Fabaceae	Pitagola	Thainongpai	T	Sd	Bronchitis (4), whooping cough (8), anthelmintic (11)	The powdered seed is applied in bronchitis and whooping cough. The seed oil is taken as an anthelmintic.	23	3	1●2●3●4●5●6●7●8●9●10●11●12●13●14●15●16●17●18●19●20●21●22●23●24●25●26●27●28●29●30●31●32●33●34●35●36●37●38●39●40●41●42●43●
*Dioscorea bulbifera* L. (bot-t1019), Dioscoreaceae	Metealu	Ram bara	C	Tb	Anthelmintic (21)	Boiled tubers are taken as an anthelmintic.	21	1	1●2●3●4●5●6●7●8●9●10●11●12●13●14●15●16●17●18●19●20●21●22●23●24●25●26●27●28●29●30●31♣32●33●34●35●36▲37●38●39●40●41▲42●43●
*Dioscorea hispida* Dennst. (bot-t1047), Dioscoreaceae	Loma alu	Chaiaibu	C	L	Dog bites (9), fever (14)	A paste prepared from the leaves is used to treat dog bites and fever.	23	1	1●2●3●4●5●6●7●8●9●10●11●12●13●14●15●16●17●18●19●20●21●22●23●24●25●26●27●28●29▲30●31●32●33●34●35●36●37●38●39●40●41●42●43●
*Drimia indica* (Roxb.) Jessop (bot-t10107), Asparagaceae	Ban piaj	Sommulung	H	Bb	Cough (5), asthma (9)	A decoction of the bulb is taken at a dose of about 100 ml per day for 10 days in asthma. A paste of the bulb is taken with honey to treat cough.	14	2	1●2●3●4♣5●6●7●8●9●10▲11●12●13●14●15●16●17●18●19●20●21●22●23●24●25●26●27●28●29●30●31●32●33●34●35●36●37●38●39●40●41●42●43●
*Elaeocarpus floribundus* Blume (bot-t1088), Elaeocarpaceae	Jalpai	Anuran	T	Fr,Sd	Skin disease (4), rheumatism (3), cough (6) dysentery and diarrhea (10)	The fruit is taken to treat dysentery and diarrhea. The seed oil is used to lessen inflammation due to rheumatism. The seed oil is also used to treat various skin diseases. In addition, the warm seed oil is used to massage the chest of children to treat cough.	23	5	1●2●3●4♣5●6●7●8●9●10●11●12●13●14●15●16●17●18●19●20●21●22●23●24●25●26●27●28●29●30●31●32●33●34●35●36●37●38●39●40●41●42●43●
*Entada rheedii* Spreng. (bot-t1072), Mimosaceae	Gilla	Pai	H	R, Sd	Joint pain (9), diarrhea (13)	A seed paste is used to treat joint pain. A root extract is taken at a dose of two spoonfuls three times daily for 10 days to cure diarrhea.	22	2	1●2●3●4●5●6●7●8●9●10●11●12●13●14●15●16●17●18●19●20●21●22●23●24●25●26●27●28●29●30●31●32●33●34●35●36●37●38●39●40●41●42●43●
*Erigeron sublyratus* Roxb. ex DC (bot-t1098), Asteraceae	Binajeron	unknown	H	L	Abdominal pain (1), diarrhea, (2), cancer (2)	A decoction of leaves is taken, at a dose of about 30 ml twice daily for 3 days, for abdominal pain and cancer. Juice of the leaves is taken to treat diarrhea.	5	3	1●2●3●4●5●6●7●8●9●10●11●12●13●14●15●16●17●18●19●20●21●22●23●24●25●26●27●28●29●30●31●32●33●34●35●36●37●38●39●40●41●42●43●
*Ficus hispida* L.f. (bot-t1066), Moraceae	Dumur	Themaset	T	Fr	Jaundice (8), fever (8), tumor (7)	Fruits are cooked with other (unknown) ingredients and consumed as vegetables, taking as much as possible for 1 month to treat jaundice and fever. A paste of the fruits is smeared to treat tumors.	23	1	1●2●3●4●5●6●7●8●9●10▲11●12●13●14●15●16●17●18●19●20▲21●22●23●24●25●26●27▲28●29●30●31●32●33●34●35●36▲37●38▲39●40●41▲42●43●
*Ficus religiosa* L. (bot-t1085), Moraceae	Pan bat	Robang	T	L	Skin disease (14)	Leaves are used to treat skin diseases.	14	1	1●2●3●4●5●6●7●8●9●10●11●12●13●14●15●16●17●18●19●20●21●22●23♣24●25●26●27●28●29●30●31●32●33♣34●35●36▲37♣38●39●40●41●42●43●
*Foeniculum vulgare* Mill. (bot-t1036), Apiaceae	Mouri	Deinak	H	Sd	Carminative (19)	A paste prepared from the seeds is taken as a carminative.	19	1	1●2●3●4●5●6●7●8●9●10●11●12●13●14●15●16●17●18●19●20●21●22●23●24●25●26●27●28●29●30●31●32●33●34●35●36●37●38●39●40●41●42●43●
*Gardenia coronaria* Buch.-Ham. (bot-t1076), Rubiaceae	Bankamal	Molaihang	T	L	Rheumatic pain (7)	An extract of the leaves is used for the treatment of rheumatic pain.	07	1	1●2●3●4●5●6●7●8●9●10●11●12●13●14●15●16●17●18●19●20●21●22●23●24●25●26●27●28●29●30●31●32●33●34●35●36●37●38●39●40●41●42●43●
*Glinus oppositifolius* (L.) Aug. DC. (bot-t1086), Molluginaceae	Gima	Bacchain	H	L	Skin disease (17)	Leaf juice is taken at a dose of two teaspoonfuls twice daily for 7 days to treat skin diseases.	17	1	1●2●3●4●5●6●7●8●9●10●11●12●13●14●15●16●17●18●19●20●21●22●23●24●25●26●27●28●29●30●31●32●33●34●35●36●37●38●39●40●41●42●43●
*Gomphrena globosa* L. (bot-t1099), Amaranthaceae	Botamphul	Meilingper	H	Rz	Diarrhea (19)	A decoction of the rhizome is used to treat diarrhea.	19	1	1●2●3●4●5●6●7●8●9●10●11●12●13●14●15●16●17●18●19●20●21●22●23●24●25●26●27●28●29●30●31●32●33●34●35●36●37●38●39●40●41●42●43●
*Grewia nervosa* (Lour.) Panigrahi (bot-t1062), Malvaceae	Assorgular	Hasalcong	S	L	Jaundice (23)	A decoction of the leaves is mixed with other (unknown) substances and honey, and is taken at a dose of one cupful daily to treat jaundice.	23	1	1●2●3●4●5●6●7●8●9●10●11●12●13●14●15●16●17●18●19●20●21●22●23●24●25●26●27●28●29●30●31●32●33●34●35●36●37●38●39●40●41●42●43●
*Helianthus annuus* L. (bot-t1068), Asteraceae	Surjamuki	Namheiper	H	L, Sd	Malaria (9), coughs and colds (24)	An extract of leaves is taken at a dose of one teaspoonful three times daily after meals for 2 months to treat malaria. A paste of the seeds is used to treat coughs and colds.	33	3	1●2●3●4●5●6●7●8●9●10●11●12●13●14●15●16●17●18●19●20●21●22●23●24●25●26●27●28●29●30●31●32●33●34●35●36●37●38●39●40●41●42●43●
*Hibiscus rosa-sinensis* L. (bot-t1073), Malvaceae	Jaba	Sendopui	T	F	Piles (15), leucorrhoea (7)	A paste of the flowers is used in piles. A decoction of the flowers is taken at a dose of one cupful twice daily until cure of leucorrhoea is observed.	22	2	1●2●3▲4●5▲6●7▲8♣9●10▲11●12**▲**13●14●15●16●17●18●19▲20▲21●22●23●24●25●262▲7●28●29●30●31●32●33♣34●35●36●37●38●39●40▲41●42▲43●
*Hygrophila difformis* (l.f.) Blume (bot-t10117), Acanthaceae, least concern	Filamish	Unknown	*H*	WP	To increase sexual desire (4)	A decoction of the plant is taken to increase sexual desire.	4	1	1●2●3●4●5●6●7●8●9●10●11●12●13●14●15●16●17●18●19●20●21●22●23●24●25●26●27●28●29●30●31●32●33●34●35●36●37●38●39●40●41●42●43●
*Hyptis suaveolens* (L.) Poit. (bot-t1093), Lamiaceae	Tokma	Biparthu	H	Sd, L	Tumor (6), constipation (9)	A soft drink prepared from the seeds is taken, consuming as much as possible to treat constipation. The leaf juice is taken at a dose of two teaspoonfuls daily for 10 days for the treatment of tumors.	15	2	1●2●3●4●5▲6▲7●8●9▲10▲11▲12**▲**13▲14●15●16▲17●18●19●20▲21●22**▲**23●24▲25●26▲27●28●29●30●31●32●33●34●35●36●37●38●39●40●41●42●43●
*Imperata cylindrica* (L.) Raeusch. (bot-t10100), Poaceae	Ulu	Lieloang	*H*	R	Fever and cough (6)	A decoction of the root with honey is taken at a dose of one teaspoonful twice daily for 3 days, to cure fevers and cough.	6	2	1●2●3●4●5●6●7●8●9●10●11●12●13●14●15●16●17●18●19●20●21●22●23●24●25●26●27●28●29●30●31●32●33●34●35●36●37**▲**38●39●40●41●42●43●
*Ipomoea mauritiana* Jacq. (bot-t1082), Convolvulaceae	Bhuikumra	Ratrui	H	Tb	Sexual disabilities (18)	Tubers are used for the treatment of sexual disabilities.	18	1	1●2●3●4●5●6●7●8●9●10●11●12●13●14●15●16●17●18●19●20●21●22●23●24●25●26●27●28●29●30●31●32●33●34●35●36●37●38●39●40●41●42●43●
*Jasminum sambac* (L.) Aiton (bot-t1087), Oleaceae	Beli	Thenerper	S	F	Skin disease (20), asthma (14)	A paste prepared from the flowers is applied in skin disease. A decoction of flowers with mustard oil is taken in asthma.	34	2	1●2●3●4●5●6♣7●8●9●10●11●12●13●14●15●16●17●18●19●20●21●22●23●24●25●26●27●28●29●30●31●32●33●34●35●36●37●38●39●40●41●42▲43●
*Jasminum scandens* (Retz.) Vahl (bot-t1038)	Ban jui	Chilokong	S	L	Conjunctivitis (12)	Two to three drops of the leaf extract are dropped into the eyes for the treatment of conjunctivitis.	12	1	1●2●3●4●5●6●7●8●9●10●11▲12●13●14●15●16●17●18●19●20●21●22●23●24●25●26●27●28●29●30●31●32●33●34●35●36●37●38●39●40●41●42●43●
*Justicia adhatoda* L. (bot-t1028), Acanthaceae	Basak	Tumpang	S	L	Bronchitis (6), high blood pressure (10)	The leaf extract is taken at a dose of three teaspoonfuls once daily for 5 days, to treat bronchitis and high blood pressure.	16	2	1●2●3▲4♣5▲6▲7▲8●9●10▲11♣12**▲**13**▲**14**▲**15●16▲17▲18●19▲20▲21●22●23●24▲25●26▲27●28●29●30●31●32●33♣34▲35●36●37●38●39●40●41▲42▲43▲
*Lablab purpureus* (L.) Sweet (bot-t1051), Fabaceae	Shim	Barui	C	L	Eczema (19), ringworm (14)	A paste of the leaves is applied to the affected areas of eczematous skin. The same formulation along with honey is also applied to ringworm.	33	2	1●2●3●4♣5●6●7●8●9●10●11●12●13●14●15●16●17●18●19●20●21●22●23●24●25●26●27●28●29●30●31●32●33●34●35●36●37●38●39●40●41●42●43●
*Lasia spinosa* (L.) Thwaites (bot-t1074), Araceae, least concern	Kantakachu	Manitri	H	L	Piles (19), bone fractures (4)	An extract of the leaves is taken at a dose of one cupful daily for 15 days to treat piles. A paste of the leaves is used to treat fractures of bone.	23	2	1●2●3♣4●5●6●7●8●9●10●11●12●13●14●15●16●17●18●19●20●21●22●23●24●25●26●27●28●29●30●31●32●33●34●3536●37●38●39●40●41●42●43● ●
*Mikania micrantha* Kunth (bot-t1041), Asteraceae	Toopainna lata	Belnum	H	L	Cuts and wounds (5), dysentery (5), gastric ulcers (4), dyspepsia (3), hemorrhage (1)	The leaves are used in dysentery and gastric ulcers. A decoction of the leaves is considered useful in dyspepsia. Crushed fresh leaves are applied to cuts and wounds to stop hemorrhages.	18	6	1●2●3●4●5●6▲7●8●9●10●11●12●13●14●15●16●17●18●19●20●21●22●23●24●25●26●27●28●29●30●31●32●33●34●35●●36●37●38♣39●40♣41●42●43●
*Mimosa pudica* L. (bot-t1075), Mimosaceae, least concern	Lajjabati	Beljak	U S	R	Piles (8), dysentery (4)	An extract prepared from the root is taken twice a day for 1 month to treat piles and dysentery.	12	2	1♣2▲3●4▲5▲6▲7▲8●9♣10▲11●12**▲**13●14●15▲16●17▲18●19▲20♣21▲ 22♣23●24●25●26▲27●28●29●30▲31●32●33♣34●35●36●37●38▲39♣40♣41●42●43♣
*Miscanthus fuscus* (Roxb.) Benth (bot-t10118), Poaceae	Fusca	Champuither	H	R	Cancer (4), cough (7)	A decoction of the root, along with unknown ingredients, is taken at a dose of one cupful twice daily to treat cancer. An extract of the root is directly taken to treat cough.	11	2	1●2●3●4●5●6●7●8●9●10●11●12●13●14●15●16●17●18●19●20●21●22●23●24●25●26●27●28●29●30●31●32●33●34●35●36●37●38●39●40●41●42▲43●
*Momordica charantia* L. (bot-t1022), Cucurbitaceae	Kakrol	Vurluk	H	Fr	Anthelmintic (7), dysentery (10), fever (6), jaundice (6), pneumonia (3)	The fruit is taken as a curry, which is useful in dysentery, fever and jaundice. The leaves are used as an anthelmintic. The young leaves are eaten as leafy vegetables to treat jaundice and pneumonia.	32	5	1●2♣3▲4●5●6●7▲8●9●10●11●12●13●14●15▲16●17●18●19●20▲21●22●23●24●25♣26●27●28●29●30●31●32●33●34●35●36●37●38♣39●40●41●42♣43▲
*Nicotiana tabacum* L. (bot-t1091), Solanaceae	Tamak	Bilao	H	L	Toothache (14), stimulant (20)	A powder prepared from the dry leaves is applied to the affected area as a remedy for toothache and also used as stimulant.	34	1	1●2●3●4●5●6●7●8●9●10▲11●12●13●14●15●16●17●18●19●20●21●22●23●24●25●26●27●28●29●30●31●32●33▲34●35●36●37●38●39●40●41●42●43●
*Ocimum basilicum* L. (bot-t1043), Lamiaceae	Babui Tulsi	Voiperfu	H	L	Bronchitis (5), diarrhea and dysentery (18)	The leaves are used in curries as an additive for aroma. This species is planted in home gardens for its pleasant smell and also as an ornamental. The leaves are used for the treatment of diarrhea, dysentery and bronchitis.	22	3	1♣2●3▲4●5●6●7●8●9●10●11●12●13●14●15●16▲17●18▲19●20▲21▲22●23●24●25●26●27●28▲29●30●31●32●33●tpins34●35●36●37●38●39●40●41▲42●43●
*Ocimum sanctum* L. (bot-t1039), Lamiaceae	Tulsi	Voiperfu	H	L	Cough and colds (15)	A paste prepared from the leaves is used to treat coughs and colds.	15	2	1●2●3●4●5●6●7●8♣9♣10●11●12●13●14●15♣16●17♣18●19●20♣21●22♣23●24●25●26●27♣28●29●30●31●32●33●34●35●36●37●38●39●40●41●42▲43▲
*Operculina turpethum* (L.) Silva Manso (bot-t1020), Convolvulaceae	Dudh kalmi	Kainem	C	R	Anthelmintic (10)	The root extract is taken as an anthelmintic.	10	1	1●2●3●4●5●6●7●8●9●10●11●12●13●14●15●16●17●18●19●20●21●22●23●24●25●26●27●28●29●30●31●32●33●34●35●36●37●38●39●40●41●42●43●
*Oryza sativa* L. (bot-t1067), Poaceae	Dhan	Chang	H	Sd	Malaria (11), abdominal pain (12)	A beer prepared from rice is taken to prevent malaria. In addition, the aqueous liquor from steeping rice in water overnight is taken to treat abdominal pain.	23	2	1●2●3●4●5●6●7●8●9●10●11●12●13●14●15●16●17●18●19●20●21●22●23●24●25●26●27●28●29●30●31●32●33●34●35●36●37●38●39●40●41●42●43●
*Ottelia alismoides* (L.) Pers (bot-t1093), Hydrocharitaceae, least concern	Panicola	Unknown	H	WP	Eye disease (7), tuberculosis (5), tumor (6)	Whole plants are wrapped in banana leaves and heated for 10 min. An extract of the prepared plant is poured (2–3 drops) into the eyes, to treat eye disease. A decoction of the whole plant is taken (as much as possible) to treat tuberculosis. A paste of the plant, along with leaves of *Paederia foetida*, is smeared on tumors.	18	3	1●2●3●4●5●6●7●8●9●10●11●12●13●14●15●16●17●18●19●20●21●22●23●24●25●26●27●28●29●30●31●32●33●34●35●36●37●38●39●40●41●42●43●
*Phyllanthus emblica* L. (bot-t1054), Euphorbiaceae	Amloki	Choalu	T	Fr	Gastritis (5), high blood pressure (4)	Fruits are eaten to treat gastritis and high blood pressure.	9	2	1●2▲3●4♣5●6♣7♣8▲9●10●11**▲**12♣13●14●15♣16▲17♣18●19▲20♣21●22**▲**23●24▲25●26▲27●28●29●30●31●32●33●34●35●36▲37♣38●39●40●41●42▲43●
*Piper betel* L. (bot-t1061), Piperaceae	Pan	Panthongna	C	L	Indigestion (2), colic (1), diarrhea (2), headache (2), masticatory substance (1), stimulant (1)	Leaves are used for the treatment of indigestion, colic, diarrhea and headache. Leaves are also used as a masticatory substance and stimulant.	9	6	1●2●3●4●5●6●7▲8●9▲10●11▲12**▲**13●14●15**▲**16●17●18●19●20●21●22●23●24●25●26●27▲28●29●30●31●32●33●34●35●36●37●38●39●40●41●42●43●
*Portulaca oleracea* L. (bot-t1092), Portulacaceae	Nune	Bakchen	H	Sd	Toothache (8), asthma (12)	The fried seed paste is used for toothache and asthma.	20	2	1▲2●3●4●5●6●7●8●9●10●11●12●13●14●15●16●17●18●19●20●21●22●23●24●25●26●27●28●29●30●31●32●33●34♣35●36●37●38●39●40●41●42●43●
*Psidium guajava* L. (bot-t1042), Myrtaceae	Peyara	Kainem	T	B, Fr	Diarrhea and dysentery (22)	Green and ripe fruits are eaten to cure diarrhea. A decoction of the bark is used in dysentery.	22	2	1♣2●3●4●5♣6●7♣8♣9●10♣11▲12♣13●14●15♣16●17●18●19●20●21●22♣23●24●25●26▲27●28●29●30●31●32●33▲34●35●36●37●38●39●40●41●42♣43●
*Saraca asoca* (Roxb.) Willd. (bot-t10102), Fabaceae, vulnerable	Asok	Licung	T	B	Diarrhea (6), leucorrhoea (4)	A decoction prepared from the bark, along with leaves of *Centella asiatica*, is taken orally at a dose of one cupful twice daily for 7 days to treat leucorrhoea. Juice of the bark is taken in diarrhea.	10	2	1●2●3●4●5●6●7▲8●9▲10●11●12●13●14●15●16●17●18●19●20▲21●22●23●24●25●26●27●28●29●30●31●32●33♣34●35●36●37●38●39●40●41●42●43●
*Senna alata* (L.) Roxb. (bot-t1078), Caesalpiniaceae	Dadmordan	Pailang	S	L	Ringworm and eczema (16)	The leaf juice is taken to treat ringworm, while young leaves are used to treat eczema.	16	2	1●2●3●4●5●6♣7♣8♣9●10♣11♣12♣13●14●15♣16♣17●18♣19♣20♣21●22♣23●24●25●26●27●28●29●30●31●32●33●34●35●36●37♣38●39●40♣41♣42♣43●
*Senna occidentalis* (L.) Link (bot-t1063), Caesalpiniaceae	Baro Kalkasunda	Kalbeia	U S	L	Jaundice (10), malaria (16)	The juice of the leaves is taken once daily for 3 days, along with beet salt, to treat jaundice and malaria.	26	2	1●2●3●4▲5●6●7●8●9●10●11●12●13●14●15●16●17●18●19●20●21●22●23●24●25●26●27●28●29●30▲31●32●33▲34●35●36●37●38●39●40●41●42●43●
*Sesbania sesban* (L.)Merr. (bot-t1021), Fabaceae	Dhaincha	Sendopui	T	L	Anthelmintic (6), colds (5)	The juice of the fresh leaves is used as an anthelmintic and to treat colds.	11	2	1●2●3●4●5●6●7●8●9●10●11●12●13●14●15●16●17●18●19●20●21●22●23●24●25●26●27●28●29●30●31●32●33●34●35●36●37●38●39●40●41●42●43●
*Smilax ovalifolia* Roxb.ex D.Don (bot-t1094), Smilacaceae	Kumarilata	Voishisong	C	St, L	Ulcer (11)	An extract of leaves and stems is mixed with black pepper and taken three times daily to treat ulcers.	11	1	1●2●3●4●5●6●7●8●9●10●11●12●13●14●15●16●17●18●19●20●21●22●23●24●25●26●27●28●29●30●31●32●33●34●35●36●37●38●39●40●41●42●43●
*Solanum torvum* Sw. (bot-t1056), Solanaceae	Titbagun	Anchangti	S	Fr	Gastritis (16), fever (5)	Unripe fruits are cooked as vegetables and are taken for the treatment of gastritis and fever.	21	2	1●2●3●4●5●6●7♣8♣9●10♣11●12●13●14●15●16●17●18●19●20●21●22♣23●24●25●26▲27●28●29●30●31●32●33●34●35●36▲37▲38●39●40♣41●42●43●
*Spilanthes acmella* (L.) L. (bot-t1081), Asteraceae	Marhatitiga	Ankasa	H	WP	Scabies (10), colic (7)	A paste prepared from the whole plant is used to treat scabies and colic.	17	1	1●2●3●4●5●6▲7●8●9●10●11●12●13●14●15●16●17●18●19●20●21●22●23●24●25●26●27●28●29●30●31●32●33●34●35●36●37●38●39●40●41●42●43●
*Spondias pinnata* (L.f.) Kurz (bot-t1077), Anacardiaceae	Amra	Thaipial	T	Fr	Rheumatism (10), sore throat (5)	Fruits are eaten. The unripe fruit is useful for rheumatism and sore throat.	15	2	1●2●3●4●5●6▲7●8●9▲10●11●12**▲**13●14●15●16●17●18●19●20●21●22●23●24▲25●26●27●28●29●30●31●32●33●34●35●36▲37●38●39●40●41●42▲43●
*Sterculia villosa* Roxb. (bot-t1095), Malvaceae	Udal	Guiza	T	L	Urinary problems (22)	Leaf juice is taken early in the morning for relief of urinary problems.	22	1	1●2●3▲4●5●6●7●8●9●10●11▲12●13●14●15●16▲17▲18●19●20●21●22▲23●24●25●26●27●28●29●30●31●32●33●34●35●36●37●38▲39●40●41▲42●43●
*Syzygium cumini* (L.) Skeels (bot-t1023), Myrtaceae	Jam	Inmui	T	B, Fr	Anthelmintic (10), blood dysentery (12)	Bark is used as an anthelmintic and for blood dysentery. Ripe fruits are eaten as an anthelmintic.	22	2	1●2♣3●4●5♣6●7●8●9▲10●11●12♣13●14●15●16●17●18●19▲20●21●22●23●24●25●26●27●28●29●30●31●32●33●34●35●36●37●38●39●40●41●42●43●
*Syzygium fruticosum* DC. (bot-t10103), Myrtaceae		Inmui	T	L	Blood dysentery (13)	A paste of the leaves is given at a dose of one teaspoonful three times daily to treat blood dysentery.	13	1	1●2●3●4♣5●6●7●8●9●10●11●12●13●14●15●16●17●18●19●20♣21●22●23●24●25●26●27●28●29●30●31●32●33●34●35●36●37●38●39●40●41●42●43●
*Tagetes erecta* L. (bot-t1040), Asteraceae	Genda	Darken	H	L	Haemostatic (9)	The leaf paste is applied to fresh cuts to stop bleeding.	9	1	1●2●3▲4♣5●6●7●8●9●10●11●12●13●14●15●16●17●18●19●20●21●22●23●24●25●26●27●28●29●30●31●32●33●34●35●36●37●38♣39●40●41●42●43●
*Tamarindus indica* L. (bot-t10105), Fabaceae	Tatul	Thenthela kung	T	Fr	Cough (7), dysentery and diarrhea (27)	A fruit decoction is used orally to treat diarrhea, dysentery and cough.	34	3	1●2▲3●4▲5▲6●7▲8●9▲10●11●12♣13●14▲15▲16●17●18●19●20▲21●22♣23●24●25●26●27●28●29●30▲31●32●33●34●35●36●37●38♣39●40●41●42●43●
*Tectona grandis* L.f. (bot-t1030), Lamiaceae	Segun	Sagunkung	T	St, B	Eczema (10), ringworm (14), diarrhea (10)	An oily product from stem chips is used in eczema and ringworm. The bark is considered useful in ringworm and diarrhea.	34	3	1●2●3●4●5●6●7▲8●9●10●11●12●13●14●15●16●17●18●19●20●21●22●23●24●25●26●27●28●29●30●31●32●33●34●35●36●37●38●39●40●41●42●43●
*Tephrosia purpurea* (L.) Pers. (bot-t1064), Fabaceae	Bannil	Bairi	H	L, Sd	Jaundice (11), scabies (3), eczema (10), skin diseases (8), tuberculosis (2),	The leaves are used in the treatment of jaundice and tuberculosis. The seed oil is used to treat scabies, eczema and other skin diseases.	34	5	1●2●3●4●5●6●7●8●9●10●11●12●13●14●15●16●17●18●19●20●21●22♣23●24●25●26●27●28●29●30●31●32●33●34●35●36●37●38●39●40●41●42●43●
*Terminalia chebula* Retz. (bot-t1057), Combretaceae	Haritaki	Sikabu	T	Fr	Gastritis (5), pain during menstruation (2), asthma (3), bronchitis (2)	Ripened green fruits are taken for the treatment of gastritis. A decoction of fruits with honey is taken at a dose of one teaspoonful three times daily in asthma and bronchitis. A paste of the fruit is smeared around the vaginal area to give relief from pain during menstruation.	12	4	1●2▲3●4♣5▲6▲7▲8▲9●10●11●12**▲**13♣14▲15▲16●1718●19●20▲21●22▲23▲24▲25●26▲27●28●29●30●31●32●33▲34●35●36●37♣38▲39●40▲41●42♣43●
*Trevesia palmata* (Roxb.ex Lindl.) Vis. (bot-t1089), Araliaceae	Argoja	Munia vanghem	T	R, Fr	Snakebite (15)	A paste prepared from the root and fruits is applied to snakebite.	15	1	1●2●3●4●5●6●7●8●9●10●11●12●13●14●15●16●17●18●19●20●21●22●23●24●25●26●27●28●29●30●31●32●33●34●35●36●37▲38●39▲40▲41●42●43●
*Urena lobata* L. (bot-t10106), Malvaceae	Jangli ghagra	Coptrit	S	F	Cough (20), fever (12)	Juice of the flowers, with mustard oil, is taken to treat cough and fever.	32	2	1●2●3●4●5●6●7●8●9●10▲11●12●13●14●15●16●17●18●19▲20●21●22▲23●24●25●26●27●28●29●30●31▲32▲33●34●35●36●37▲38▲39●40●41♣42●43▲
*Urginea indica* Kunth (bot-t1027), Liliaceae	Ban piaj	Sommulung	H	Bb	Asthma (6), dysentery (15)	The bulb extract is used for the treatment of asthma and dysentery.	21	2	1●2●3●4●5●6●7●8●9●10●11●12●13●14●15●16●17●18●19●20●21●22●23●24●25●26●27●28●29●30●31●32●33●34●35●36●37●38●39●40●41●42●43●
*Zingiber officinale* Roscoe (bot-t1014), Zingiberaceae	Ada	Aaithing	H	Rz, L	Food additive (1), stimulant (1), abdominal problems (1), laxative (1), dyspepsia (1), dysentery and vomiting (3), coughs, bronchitis, asthma and tuberculosis (5)	The rhizome is used as a spice, while leaves are used as an additive, stimulant, for abdominal problems and as a laxative. An infusion of the rhizome is used in dyspepsia, cough, bronchitis, asthma, dysentery, vomiting and tuberculosis.	13	11	1●2●3●4♣5♣6♣7●8●9♣10♣11**▲**12♣13●14▲15♣16●17●18●19●20♣21●22♣23●24●25●26●27●28●29●30●31●32●33●34●35●36●37♣38●39●40●41●42♣43●

Legend: *C* climber, *H* herb, *S* shrub, *T* tree, *US* under shrub, *B* bark, *Fr* fruit, *Bb* bulb, *F* flower, *L* leaves, *Sd* seed, *St* stem, *Rz* rhizome, *R* root, *Tb* tuber, *WP* whole plant, *FI* frequency of informants, *FC* frequency of citation, *UR* use report

^♣^similar use, ^▲^dissimilar use, ^●^use not reported

1 = [27]; 2 = [44]; 3 = [10]; 4 = [36]; 5 = [8]; 6 = [35]; 7 = [34]; 8 = [3];9 = [42]; 10 = [7]; 11 = [9]; 12 = [8]; 13 [49]; 14 = [50]; 15 = [51]; 16 = [39]; 17 = [40]; 18 = [52]; 19 = [11]; 20 = [12]; 21 = [53]; 22 = [13]; 23 = [15]; 24 = [54]; 25 = [47]; 26 = [55]; 27 = [56]; 28 = [57]; 29 = [58]; 30 = [59]; 31 = [41]; 32 = [43]; 33 = [45]; 34 = [60]; 35 = [61]; 36 = [62]; 37 = [63]; 38 = [64]; 39 = [65]; 40 = [66]; 41 = [67]; 42 = [68]; 43 = [69]

### Demography of informants

A total of 218 people, including traditional healers and other community members, mostly the elderly men and women, with ages ranging from 27 to 86 years were interviewed, and of them, the majority (65.6%) belonged to the age group of 51–70. We considered as informants those reporting one or more ethnomedicinal uses of a species (see Additional file 1 as an example). Demographics by gender, age, education, and occupation of participants are summarized in Table 2. Detailed clarification of informants is presented in an additional file (see Additional file 2).

**Table 2:** Demographics of the Informants

Variable	Categories	Percentage
Gender	Male	66.97
	Female	33.03
Age group	< 30	9.63
	30–50	16.51
	51–70	65.60
	70>	8.26
Education	Illiterate	45.87
	Primary	30.73
	High school	22.02
	University	1.38
Profession	Daily laborer	28.44
	Farmer	47.71
	Professional healer	6.88
	Other	16.97

### Ethnomedicinal plants and part(s)

**Fig. 2:**
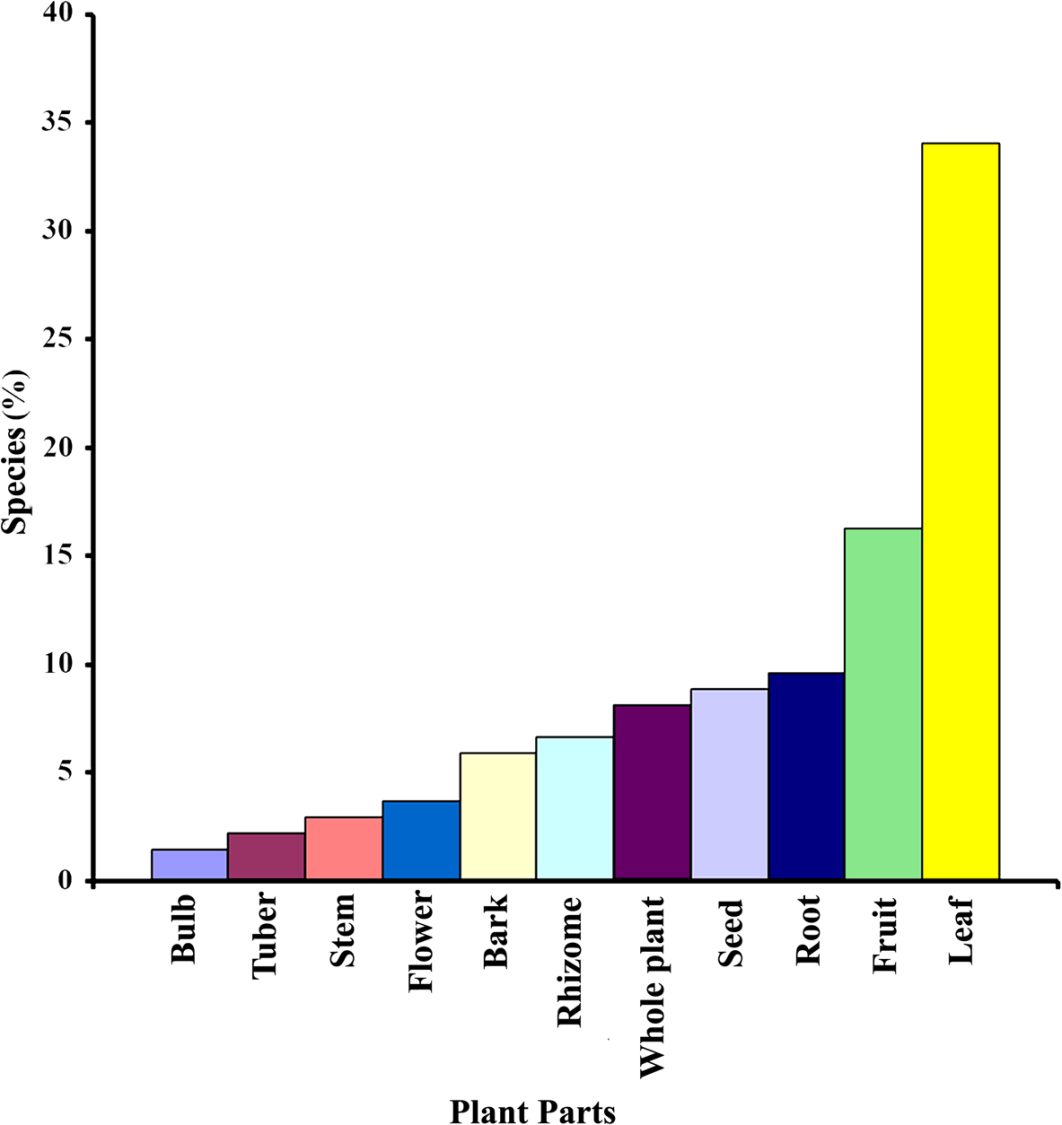
Plant parts used for the preparation of herbal medicines

The present investigation details 117 species of ethnomedicinal plants distributed across 104 genera and belonging to 54 families (Table 1). The highest numbers of ethnomedicinal plants recorded were from the Fabaceae (12 species). The second largest used families represented were the Asteraceae and Zingiberaceae (10 species each), followed by the Lamiaceae (5), Caesalpiniaceae (4), and Amaranthaceae, Apiaceae, Cucurbitaceae, and Poaceae having 3 species each. The remainder of families was represented by two or one species. However, most of these families are documented to contain active constituents and feature in different traditional systems of medicine. Of all recorded species, herbs (55 species) were found to account for the greatest number, followed by trees (35 species), shrubs (13 species), climbers (10 species), and under-shrubs (4 species). Different parts of ethnomedicinal plants are used in herbal formulations by local traditional healers for the treatment of different ailments. Among such plant parts, leaves (34.07%) were found to be the most frequently used for the preparation of herbal drugs, followed by other parts (Fig. 2).

Considering the mode of preparation of traditional medicines by the Pangkhua community, the range of methods reported for various species included decoctions, juices, extracts, pastes, powders, infusions, oils, and the use of fresh plant parts. Among these, the most common formulations were decoctions (25.93%) and fresh plant parts (23.46%), followed by juices (16.05%), pastes (14.81%), extracts (13.58%), oils (3.70%), and infusions and powders (1.23% each). Decoctions are often the most commonly encountered preparation method reported [26–30]. In some cases, processing involved drying of the plant material followed by grinding into a fine powder. Water was most commonly used if a solvent was required, with cow’s milk or honey sometimes used as a matrix or as an adjuvant to increase viscosity. Within the study community, plant medicines were usually administrated orally. Bathing in a decoction or rubbing and massaging using the plant parts were also encountered.

### Conservation status

The conservation status of all recorded plant species was checked using the International Union for Conservation of Nature (IUCN) Red List of Threatened Species [31]. A total of 12 species, namely *Acorus calamus*, *Amorphophallus paeoniifolius*, *Ammania multiflora*, *Azolla pinnata*, *Breonia chinensis*, *Centella asiatica*, *Cyperus rotundus*, *Commelina diffusa*, *Hygrophila difformis*, *Lasia spinosa*, *Mimosa pudica*, *and Ottelia alismoides* were recorded as “of Least Concern,” while only one species (*Saraca asoca*) was recorded as “vulnerable,” with all other species not included on the list.

### Quantitative analysis

The present study records the use of ethnomedicines to treat 11 categories of ailments. Of these, the most common uses were for digestive system disorders (49 species), followed by respiratory complaints (39 species) (Table 3). To ascertain the level of agreement among the informants of the Pangkhua community regarding the use of plants to treat certain disease categories, FIC values were determined. The FIC values are presented in Table 3. It is clear that the FIC values showed variation, varying from 0.50 to 0.66. In the treatment of digestive system disorders, the highest FIC value (0.66) was encountered, with 141 use-reports for 49 plant species. This was followed by plants used to treat respiratory system disorders (FIC = 0.64) and so on (Table 3). In contrast, the least agreement (FIC = 0.50) between informants regarding therapeutic uses was observed for plants used to treat urinary disorders. The calculated JI indices (Table 4) ranged from 1.65 to 33.00. The highest degree of similarity was found with a study conducted in Bangladesh, while the lowest degree of similarity was found with a study conducted in Pakistan.

**Table 3:** Categories of ailments and Informant Consensus Factors (FIC)

Use category. In brackets, local name of illness used by the informants and local people	Number of taxa (Nt)	Number of use reports (Nur)	Informant consensus factor
Digestive system disorders: diarrhea (*patla paikhana*), gastritis (*gastic*, *petod gas*), dysentery (*bikar*, *amasha*), carminative (*hozmi*, *petod gas komibo*), digestive (hozomi), ulcer (*petod gha*), vomiting (*bomi*), indigestion (*hozom n hoile*), piles (*arsha*), constipation (*paikhana kosha*), anthelmintic (*krimir osod*), dyspepsia (*hozom n hoile*), laxative (*paikhana norom goribellai*).	49	141	0.66
Respiratory disorders: cough (*kof*), colds (*thanda*), pneumonia (*newmonia*), bronchitis (*jolkhashi*), tuberculosis (*jokkha*), asthma (*shash kosto*), whooping cough (*khungri khas*).	39	109	0.65
Cancer (*kancer*) and tumors (*tumar*)	7	18	0.64
Malaria (*maleria*)	5	11	0.60
Snake and dog bites (*hap and kutta kamor*)	4	8	0.57
Skin diseases: eczema (*kaur*), ringworm (*dad*), boils (*fura*), scabies (*chulkani*), burns (*pora*), cuts (*kata*) and wounds (*khoto/gha*).	24	55	0.57
Sexual and menstrual disorders: leucorrhoea (*shet shrab*), dampened sexual desire (*sex barabelllai*), impotence (*bondha*), pain during menstruation (*masik kale betha*), sexual disabilities (*bikolango*)	10	22	0.57
Pain: Abdominal pain (*tolpetod betha*), backache (*pit betha*), earache (*kanot betha*), headache (*matha betha*), joint pain (*girad betha*), rheumatic pain (*bater betha*), sore throat (*golat betha*), stomachache (*pedot betha*), toothache (*dat betha*).	18	39	0.55
Urinary disorders: dysuria (*proshaber somot betha*), kidney problems (*kidnir somossa*), urinary tract infections (*proshaber rastat gha*)	6	11	0.50
Jaundice (*jondis*)	7	18	0.64
General disorders: fever (*jor*), high blood pressure (*pressure bari gele*), colic (*shul betha*), stimulant (*uttejok*), conjunctivitis (*chokh utha*).	22	58	0.63

**Table 4:** Jaccard similarity index (JI) for local and neighboring countries

	Area of study	Indices				JI	Reference
		S. N.	A	B	C		
Bangladesh	Eleven districts of Bangladesh	1	104	237	13	3.96	[27]
	Dhaka	2	109	29	8	6.15	[44]
	Rangamati district	3	104	37	13	10.16	[10]
	Panchagarh district	4	88	68	29	22.83	[36]
	Garo hills of Durgapur	5	98	52	19	14.50	[8]
	Bandarban district	6	95	44	22	18.80	[35]
	Cox’s Bazar district	7	84	49	33	33.00	[34]
	Hazarikhil, Chittagong	8	102	28	15	13.04	[3]
	Madhupur Forest Area	9	96	57	17	12.5	[42]
	Bandarban district	10	85	127	32	17.78	[7]
	Chittagong Hill Tracts	11	97	126	20	9.22	[9]
	Durgapur	12	95	49	22	18.03	[8]
	Moulivibazar district	13	111	09	6	5.26	[49]
	Pabna district	14	103	15	14	13.46	[50]
	Joypurhat district	15	95	73	22	15.07	[51]
	Rangamati district	16	107	31	10	7.81	[39]
	Kalenga forest	17	107	25	10	8.20	[40]
	Rangamati district	18	113	20	04	3.10	[52]
	Bandarban district	19	106	47	11	7.74	[11]
	Sandwip Island, Chittagong	20	89	83	28	19.44	[12]
	Bandarban district	21	113	29	04	2.90	[53]
	Chittagong Hill Tracts	22	89	119	28	15.56	[13]
	Rangamati district	23	114	12	03	2.44	[15]
	Bandarban district	24	110	47	07	4.67	[54]
	Six districts of Northern region	25	114	21	03	2.27	[47]
	Sylhet district	26	98	55	19	14.18	[55]
	Natore district	27	111	14	06	5.04	[56]
	Kurigram district	28	112	26	05	3.75	[57]
Neighboring countries	Satpuda hills of India	29	111	46	6	3.97	[58]
	Uttar Pradesh, India	30	113	36	4	2.76	[59]
	Parbat district of western Nepal	31	114	129	4	1.67	[41]
	Jajarkot district, Nepal	32	114	57	3	1.79	[43]
	Shimoga district, India	33	102	70	15	9.55	[45]
	Sarban hills, Abbottabad, Pakistan	34	114	71	3	1.65	[60]
	Tribal areas, Pakistan	35	113	75	4	2.17	[61]
	Manipur, India	36	107	110	10	4.83	[62]
	Mizoram, India	37	105	147	12	5.00	[63]
	Mizoram, India	38	101	119	16	7.84	[64]
	Mizoram, India	39	114	54	03	1.82	[65]
	Western Mizoram, India	40	105	77	12	6.74	[66]
	Tripura state, India	41	106	114	11	5.26	[67]
	South district of Tripura, India	42	97	93	20	11.74	[68]
	Assam, India	43	109	31	08	6.06	[69]

Legend: *A* is the recorded number of species of the current study area *a*, *B* is the documented number of species of another study area *b*, and *C* is the number of species common to both areas *a* and *b*, and *S.N* is the serial number

### New ethnomedicinal plant species and uses

Our comparative analysis revealed that out of 117 ethnomedicinal plant species documented, 37 species had either no similar or any use (Table 1). Therefore, these species were compared with the research databases of SCOPUS, PubMed, Biomed Central, and Google Scholar, and the results showed that use of 12 of these species has heretofore been unreported in Bangladesh (Table 5), while 6 species have never been screened pharmacologically.

**Table 5:** List of new ethnomedicinal plant species and species as yet unscreened for pharmacological activity

Species used for the treatment of different ailments in other regions	Species reported for the first time with an ethnobotanical use in Bangladesh	Species not studied pharmacologically to date
• Ananas comosus • *Artocarpus heterophyllus* • *Blumea balsamifera* • *Blumea lacera* • *Bombax ceiba* **•** *Cheilocostus speciosus* • Cinnamomum tamala • *Coccinia grandis* • *Dioscorea hispida* • *Hyptis suaveolens* • *Mikania micrantha* • *Nicotiana tabacum* • *Piper betel* • *Spilanthes acmella* • *Spondias pinnata* • *Sterculia villosa* • *Tectona grandis* • Argyreia splendens	• Albizia myriophylla • Alternanthera pungens • Ammannia multiflora • Amberboa moschata • Antidesma velutinosum • Argyreia splendens • Azolla pinnata • Bauhinia scandens • Erigeron sublyratus • Hygrophila difformis • *Miscanthus fuscus* • Ottelia alismoides	• Amberboa moschata • Antidesma velutinosum • Argyreia splendens • Bauhinia scandens • Erigeron sublyratus • *Miscanthus fuscus*

## Discussion

Overall, this study revealed the traditional use of 117 plant species, distributed among 104 genera and belonging to 54 families to treat 11 categories of ailments, recorded from 218 traditional healers and elderly men and women. The highest number of species belonged to the Fabaceae; this dominancy may be due to the worldwide distribution of species from this family [32, 33] and, furthermore, that the Fabaceae constitute the second largest family in the flora of Bangladesh [2]. Similar results have been reported by other ethnobotanists [10, 27, 34] while [7] reported the Asteraceae as the largest family and the Fabaceae the third largest family in their study conducted in Bangladesh.

Herbs are naturally abundant in the study areas, which were mostly hilly and covered by a forest canopy, creating favorable conditions for their growth. Similar results were observed with other studies conducted in different regions of Bangladesh [3, 27, 34–36].

The preference for the use of leaves in the preparation of herbal medicines by the healers is likely due to the year-round availability of leaves, and the fact that they are easier to collect, store, process, and handle. Similar observations have been reported in allied studies in Bangladesh and other countries [28, 35, 37, 38]. Healers usually however prefer to use fresh plant materials instead of dry and stored ones for herbal preparations.

In the study area, digestive system disorders are common, largely due to a deficiency of pure water, especially in the dry season, coupled with a lack of awareness of its importance among those living in hilly and remote areas. Similarly, respiratory system disorders were second in occurrence, due to prevalence of smoking and chewing of leaves of *Nicotiana tabacum* with those of *Piper betel*. Analogously to our results, digestive system disorders were found to be the major ailment category in many other ethnomedicinal studies conducted in Bangladesh [7, 8, 14, 39, 40]. High FIC values also indicate that such species are worth investigating for bioactive compounds.

As discussed earlier, some medicinal plant species used by the healers of the studied community are also used by the healers of different communities in different parts of Bangladesh as well as in neighboring countries and beyond.

A total of 19 ethnomedicinal plant species which are commonly used by the indigenous communities of Bangladesh were selected and their known uses compared with our results (Table 6), to ascertain whether the Pangkhua community has any novel uses of these species. Alongside, we evaluated the phytochemical literature on these species. From our review, 11 species, namely *Acorus calamus*, *Aegle marmelos*, *Arecha catechu*, *Calotropis procera*, *Centella asiatica*, *Curcuma longa*, *Justicia adhatoda*, *Phyllanthes emblica*, *Saraca asoca*, *Terminalia chebula*, *and Zingiber officinale* have distinct uses within the Pangkhua community. For example, *Centella asiatica* is used analogously by the Marma community in Bandarban [35], the Rakhaing community in Cox’s Bazar [34], the Tripura community in Chittagong [3]; the local people in the Panchagarh [36], Garo, Hazong, and Bangalee communities in Durgapur [8]; the local people of 11 districts in Bangladesh [27]; and the ethnic people of western Nepal [41]. This species was also used differently in traditional medicine by traditional healers of Bangladesh and other countries [37, 42–45]. Interestingly, its use in one ailment, asthma, has been documented for the first time in this study. Similarly, the use of *Acorus calamus* as an anthelmintic has not been reported before, and the use of fruit of *Aegle marmelos* to treat asthma is recorded herein for the first time, while its leaves were used in combination with other plants [46]. Other unreported uses of established ethnomedicinal species include *Arecha catechu* as a carminative, *Calotropis procera* to treat asthma and snake bite, *Curcuma longa* as a laxative and to treat fever, *Justicia adhatoda* and *Phyllanthes emblica* to reduce high blood pressure, *Saraca asoca* to treat diarrhea and leucorrhea, *Terminalia chebula* to reduce pain during menstruation and to treat bronchitis, and *Zingiber officinale* as a laxative and to treat dyspepsia and tuberculosis.

**Table 6:** Comparative ethnobotanical uses of selected species among the Pangkhua and wider Bangladesh

Scientific name	Documented secondary metabolites in phytochemical studies	Ethnomedicinal application(s) among the Pangkhua^a^	Previous ethnomedicinal report in Bangladesh
*Acorus calamus*	β-asarone [70]	**Anthelmintic**, gastritis	Asthma [39, 52]; menstrual problem and bowel pain [13]; asthma and wounds [55]; eczema [35]; gastritis, vomiting and splenomegaly [34]; hair problem [10]; cough [71]; constipation, edema, and indigestion [72]; indigestion [7]
*Aegle marmelos*	Taxol [73]; 2-isopropenyl-4-methyl-1-oxa-cyclopenta[*b*]anthracene-5,10-dione and (+)-4-(2′-hydroxy-3′-methylbut-3′-enyloxy)-8H-[1,3]dioxolo[4,5-h]chromen-8-one, imperatorin, *β*-sitosterol, plumbagin, 1-methyl-2-(3′-methyl-but-2′-enyloxy)-anthraquinone, *β*-sitosterol glucoside, stigmasterol, vanillin and salicin [74]; anhydromarmeline (**1**), aegelinosides A and B [75]	Digestive, **asthma**	Constipation, peptic ulcer and respiratory disorder [8]; dysentery and indigestion [13]; dysentery and diarrhea [9]; sedative [11]; abscess, fever, dysentery and indigestion [51]; dysentery [12]; itches [50]; insomnia [35]; vomiting [34]; stomachache and blood dysentery [71]; diarrhea, dysentery, constipation, peptic ulcer and respiratory disorder [72]; digestive, dysentery and diarrhea [7]
*Aloe vera*	Dihydrocoumarin derivatives, compounds **1** and **2** [76]; *p* –coumaric acid, ascorbic acid, pyrocatechol and cinnamic acid [77]; Di(2-ethylhexyl) phthalate [78]	Burns	Piles, menstrual disease and sex problems [51]; skin disease [34]; burn and skin disease [10, 78]
*Areca catechu*	Fernenol (fern-9(11)-en-3α-ol), arundoin (fern-9(11)-en-3α-ol ME), stigmasterol and *β*-sitosterol [79]; NPF-86IA, NPF-86IB, NPF-86IIA, and NPF-86IIB [80]	**Carminative**	Diarrhea [34]; [71]
*Azadirachta indica*	Limonoids 3-deacetyl-3-cinnamoylazadirachtin, 1-tigloyl-3-acetyl-11-methoxyazadirachtinin, azadirachtin, 22,23-dihydro-23β-methoxyazadirachtin and 3-tigloylazadirachtol [81]	Scabies, malaria	Diabetes [44]; allergy, eczema, skin disease and diabetes [8]; scabies and itches [53]; diabetes [39]; cold and cough [13]; eczema [9]; worm, chicken pox, eczema, itches and helminthiasis [51]; allergy [40]; blood poisoning, itches and eczema [49]; Itches and ringworm [82]; toothache, skin disease and insecticide [12]; itches [55]; itches [50]; chicken pox and measles [35]; chicken pox, high blood pressure, gastritis, flatulence, jaundice, vomiting and malaria [34]; skin disease [10]; pain, wound healing, small pox, eczema, skin disease, fever and cough [72]; insecticide, diabetes, fever, skin diseases, piles and malaria [7]
*Calotropis gigantea*	Lupeol [83]; isorhamnetin-3-*O*-rutinoside, isorhamnetin-3-*O*-glucopyranoside and taraxasteryl acetate, isorhamnetin-3-*O*-[2-*O-*β-d-galactopyranosyl-6-*O*-α-l-rhamnopyranosyl]-β-d-glucopyranoside [84]	Rheumatic pain	Rheumatism [13]; Elephantiasis, emollient, pain, boils and abscess [54]; rheumatism [12]; wounds, paralysis and erectile dysfunction [55]; pain [50]; pain [35]; gout, toothache, rheumatic pain and catarrh [34]; rheumatism [71]; constipation, fever and stomach disorder [72]; cough, asthma and rheumatism [7]
*Calotropis procera*	5-Hydroxy-3,7-dimethoxyflavone-4′-*O*-β-glucopyranoside, 2β,19-epoxy-3β,14β-dihydroxy-19-methoxy-5α-card-20(22)-enolide and β-anhydroepidigitoxigenin-3β-*O*-glucopyranoside, along with two known compounds, uzarigenine and β-anhydroepidigitoxigenin [85]; calotroprocerol A, calotroproceryl acetate A, calotroprocerone A and calotroproceryl acetate B [86]	**Asthma**, **snake bite**	Rheumatism [13]; piles [51]; diabetes [7]
*Cassia fistula*	Catechin [87]; 1, 8- dihydroxyanthraquinone-3-carboxylic acid [88]	Jaundice, dysentery	Helminthiasis, cough and nervous weakness [8]; constipation [39]; skin disease and jaundice [13]; fever [11]; coughs, helminthiasis, diabetes, irregular urination, edema, and constipation [54]; tonsillitis, constipation and rheumatic pain [89]; constipation [52]; constipation [12]; diarrhea, dysentery and piles [34]; diarrhea [71]; dysentery and constipation [7]
*Centella asiatica*	Asiaticoside G, five triterpenoids, asiaticoside, asiaticoside F, asiatic acid, quadranoside IV, and 2α,3β,6β-trihydroxyolean-12-en-28-oic acid 28-*O*-[α-l-rhamnopyranosyl-(1→4)-β-d-glucopyranosyl-(1→6)-β-d-glucopyranosyl] ester, and four flavonoids, kaempferol, quercetin, astragalin, and isoquercetin [90]	Conjunctivitis, dysentery, impotence, **asthma**	Diabetes [44]; dysentery, wounds, burns, and skin lesion [8]; dysentery [13]; syphilis and ulcer [9]; dysentery, eczema and headache [51]; dysentery [40]; dysentery and diarrhea [89]; stomach pain and flatulency [82]; spermatorrhea [56]; dysentery and fever [12]; dysentery and intestinal dysfunction [55]; dysentery [50]; vomiting, dysentery, diarrhea and dehydration [35]; fever, pyorrhea, impotence, gastritis and jaundice [34]; pain and dysentery [71]; hypertension, wounds, burns and skin lesion [72]; fever, loss of smell and taste, carbuncles and dysentery [7]; conjunctivitis [91]
*Curcuma longa*	Curcuminoids [92] (Revathy 2011), curcumin, demethoxycurcumin, bisdemethoxycurcumin, ar-turmerone and curlone [93]	Eczema, dysentery, coughs, cold, **fever**, **laxative**	Diarrhea and flatulence [8]; joint pain and blood purifier [13] blood disease [9]; abscess and eczema [51]; bone fracture and helminthiasis [57]; pain [49]; itches and ringworm [82]; skin disease [12]; bone fracture and sex stimulant [55]; cough and eczema [34, 71]; diarrhea and flatulence [72]; scabies, malaria, chicken pox and blood purifier [7]; dysentery [94]
*Justicia adhatoda*	Vasicoline, vasicolinone, vasicinone, vasicine, adhatodine, and anisotine [95]	Bronchitis, **high blood pressure**	Intestinal disorder, pneumonia, cough, scabies and skin disease [8]; coughs [39]; cough, cold, asthma and chest pain [9]; helminthiasis, diarrhea and constipation [11]; malaria, cough and cold [54]; cold and cough [40]; cough [49]; skin infections [82]; cough [12, 34, 50, 55, 71]; skin cancer, cough and pain [35]; cough and fever [10]; cough, pneumonia and asthma [72]; asthma and cough [7]; bronchitis [96]
*Ocimum sanctum*	[16-Hydroxy-4,4,10,13-tetramethyl17-(4-methyl-pentyl)-hexadecahydro-cyclopenta[a]phenanthren-3-one [97]	Cough and colds	Diabetes [44]; fever, cold and cough [13]; cough and cold [54]; cough and fever [51]; cold and cough [40]; rheumatic pain [56]; cough and pneumonia [12]
*Phyllanthus emblica*	Gallic acid, methyl gallate, corilagin, furosin, and geraniin [98]	Gastritis, **high blood pressure**	Diabetes [44]; allergy and gastritis [8]; anemia [39]; dysentery, anemia and pain [13]; insomnia [9]; aphrodisiac, energizer and fever [54]; burning sensation, vomiting, cough, indigestion and jaundice [51]; fever and malaria [40]; bleeding from gums, loss of appetite and headache [82]; paralysis [56]; stimulant [15]; diuretic, tonic, laxative and gastritis [12]; intestinal dysfunction and blood purifier [55]; abdominal gas and fever [35]; urinary tract infection and hysteria [71]; edema, constipation and gonorrhea [72]
Saraca asoca	Lyoniside, nudiposide, 5-methoxy-9-β-xylopyranosyl-(−)-isolariciresinol, icariside E_3_, schizandriside, (−)-epicatechin, epiafzelechin-(4β→8)-epicatechin and procyanidin B_2_ [99]	**Diarrhea**, **leucorrhoea**	Irregular menstruation [12]; ulcer, piles, dyspepsia, dysentery and stomachache [72]
*Senna alata*	Kaempferol and kaempferol 3-*O*-gentiobioside [100]; 3,5,7,4-tetrahydroxy flavone [101]	Ringworm and eczema	Skin disease and scabies [8]; skin disorder and eczema [39]; skin disease [13]; eczema [9]; ringworm and eczema [11]; ringworm, eczema, itch, scabies and skin disease [54]; eczema [7, 35, 51, 71, 82]; skin infection [52]; skin disease [12]
*Solanum torvum*	Chlorogenone and neochlorogenone [102]; solanolide 6-*O*-[α-l-rhamnopyranosyl-(1→3)-*O*-β-d-quinovopyranoside], solanolide 6-*O*-[β-d-xylopyranosyl-(1→3)-*O*-β-d-quinovopyranoside], yamogenin 3-*O*-[β-d-glucopyranosyl-(1→6)-*O*-β-d-glucopyranoside] and neochlorogenin 3-*O*-[β-d-glucopyranosyl-(1→6)-*O*-β-d-glucopyranoside] [103]	Gastritis, fever	Urinary problems, sores and fever [13]; gastritis and toothache [7]
*Tamarindus indica*	Proanthcyanidins, procyanidins [104]; furfural, palmitic acid, oleic acid and phenylacetaldehyde [105]	Cough, dysentery and diarrhea	Diabetes [44]; sinusitis and chronic cold [8]; urinary problem, cold and fever [13]; burning sensation and heart disease [51]; chicken pox, boils, rheumatism and laxative [12]; respiratory difficulties and bone fracture [50]; conjunctivitis, pain and excessive menstruation [34]; constipation and jaundice [72]; constipation, loss of appetite, diarrhea, chronic fever and dysentery [106]
*Terminalia chebula*	Chebulagic acid [107]; gallic acid, punicalagin, isoterchebulin, 1,3,6-tri-*O*-galloyl-β-d-glucopyranose, chebulagic acid and chebulinic acid [108]	Gastritis, **pain during menstruation**, asthma, **bronchitis**	Diabetes [44]; constipation and vomiting [8]; purgative and cardiac disease [13]; aphrodisiac, energizer, fever and body ache [54]; constipation, indigestion, rheumatism and urinary disease [51]; fever and malaria [40]; toothache, body pain and skin diseases [49]; bleeding from gums and loss of appetite, headache [82]; stimulant [15]; constipation [12]; indigestion, vomiting, constipation and intestinal dysfunction [55]; loss of appetite [50]; sore throat and cough [35]; cough [34]; constipation, ulcer, and edema [72]; gastritis [109]; asthma, heart disease, eye disease, itches and night blindness [91]
*Zingiber officinale*	*cis*- and *trans*-6-shogaol, 8-shogaol, 10-shogaol, 12-shogaol and *syn*- and *anti*-methyl-6-shogaol, methyl-8-shogaol, methyl-10-shogaol [110]	Food additive, stimulant, abdominal problems, **laxative**, **dyspepsia**, dysentery and vomiting, coughs, bronchitis, asthma and **tuberculosis**	Cough, stomachache and gastritis [8]; stomachache [13]; allergy [9]; fever and bronchitis [51]; abdominal pain [12]; depression and itches [50]; sore throat and cough [35]; indigestion and cough [34]; cough, stomachache, gastritis and vomiting [72]; flatulence, gastritis, carminative, abdominal pain, coughs and colds [7]; edema, asthma, chest diseases, and vomiting [111]

New uses of these plants documented among the Pangkhua are highlighted in bold

To illustrate homogeneity of use or otherwise, the JI was used to compare our study with 43 previous investigations. In total, the JI was calculated for 28 regions of Bangladesh with the Cox’s Bazar district emerging as the most similar to our study area with JI = 33.00, followed by the Panchagarh, Chittagong, and Bandarban districts (JI = 22.83, 19.44, and 18.80 respectively), while the lowest JI (2.77) was found with the study conducted by Rahman [47]. The high JI may reflect that the study area is located in the same geological zone, with similar socioeconomic and cultural characteristics. On the other hand, among three neighboring countries (India, Pakistan, and Nepal), the highest similarity was found with the adjacent state of Tipura, India (JI = 11.74) while the lowest (JI = 1.65) was from Pakistan.

### Limitations of the current study

Ethnobotanical documentation constitutes field-based research. Nevertheless, the field is not always a safe environment. A majority of the indigenous communities we studied live in forest areas, and there have been security risks due to rebel movement in these areas. It is risky to carry valuable field equipment like cameras, recorders, etc. Route access was limited to foot traffic. Language barriers were encountered, as most participants did not speak the national Bangla language requiring the use of interpreters. Seasonal variation is an important factor in the collection of voucher specimens, as in the rainy season it is difficult to both access and dry the specimens, while in the dry season the aerial parts of many plants have withered, coupled with the clearing of forest areas for cultivation during that period.

Indigenous peoples are sometimes unwilling to share their knowledge of medicinal plants with others, specifically the Bangali (Bangladeshi). They maintain the secrecy of medicinal plant use because there is a belief among them that the medicines lose their efficacy if too many people know of them, and additionally, they may be conscious about economic losses [48]. There may also be resistance to allowing themselves to become the subject of study by outsiders [48]. Therefore, potential informants must be encouraged using several techniques. Firstly, emphasis must be given to help them understand that shared information will be preserved for the benefit of their children and future generations. As their children are less frequently adopting the role of healers, without documentation, much knowledge of medicinal plants may disappear forever.

### Research highlights

1. The present study revealed that the Pangkhua community depends on a variety of ethnomedicinal plants to treat various diseases.
2. Local herbalists are predominantly aging men and women, and the Pangkhua younger generation lacks interest in following the traditional role of the healer.
3. While in many cases, the plants utilized by the Pangkhua are documented in allied literature, their preparation, mode of use, and clinical indication often differ from that of other indigenous communities.
4. The information compiled herein constitutes a rich knowledge source for taxonomists, phytochemists, environmentalists, pharmacists, and allied professionals.

## Conclusions

It can be concluded that the Pangkhua indigenous community of the Rangamati district of Bangladesh possess rich ethnomedicinal knowledge, as they use many medicinal plant species in their healthcare system. The novelty of this study is that 12 ethnomedicinal plant species have been recorded with new uses, and 6 of these species have never been screened pharmacologically. The traditional plants utilized have in some cases been validated scientifically by isolation of active ingredients, thus showing that traditional remedies are an important and effective part of indigenous healthcare systems in the district. Our findings will be helpful to ethnobotanists and phytochemists for conducting research into the isolation of active principles from these species. The preservation of these plant species is the gateway toward developing efficacious remedies for treating disease. Enhancing the sustainable use and conservation of indigenous knowledge of useful medicinal plants may benefit and improve the living standards of poor people. Hence, it is necessary to document the indigenous knowledge of useful plants and their therapeutic uses before they are lost forever.

## Additional files

Additional file 1: Group interview. There were 28 Pangkhua people present while we were conducting an interview about ethnomedicinal plant usage. We considered all 28 Pangkhua people as informants, due to each person having some knowledge regarding ethnomedicines. (TIF 6452 kb)

Additional file 2: List of 218 informants in the study, alongside their demographic characteristics (traditional healers highlighted in red). The detailed descriptions of all 218 informants, including their age, sex, location, education, and occupation were documented from the studied areas. (XLSX 19 kb)

## Abbreviations

CHTs: Chittagong Hill Tracts districts
FIC: Factor of Informant Consensus
IUCN: International Union for Conservation of Nature
JI: Jaccard index

## References

[1] Yusuf M, Chowdhury J, Wahab M, Begum J: Medicinal plants of Bangladesh. Bangladesh Council of Scientific and Industrial Research*,* Dhaka, Bangladesh 1994, 192.

[2] Pasha M, Uddin S: Dictionary of plant names of Bangladesh (vascular plants). Janokalyan Prokashani Chittagong, Dhaka, Bangladesh 2013:1–434.

[3] Faruque O, Uddin SB (2011). Ethnodiversity of medicinal plants used by Tripura community of Hazarikhil in Chittagong district of Bangladesh. J Taxon Biodiv Res.

[4] Chowdhury MSH, Koike M, Muhammed N, Halim MA, Saha N, Kobayashi H (2009). Use of plants in healthcare: a traditional ethno-medicinal practice in rural areas of southeastern Bangladesh. Int J Biodivers Sci Ecosyst Serv Manag.

[5] Thomsen M, Halder S, Ahmed F: Medicinal and aromatic plant industry development. Inter-Cooperation*,* Dhaka, Bangladesh 2005.

[6] Uddin S (2014). Bangladesh ethnobotany online database.

[7] Faruque MO, Uddin SB, Barlow JW, Hu S, Dong S, Cai Q, Li X, Hu X (2018). Quantitative ethnobotany of medicinal plants used by indigenous communities in the Bandarban District of Bangladesh. Front Pharmacol.

[8] Khan MA, Islam MK, Siraj MA, Saha S, Barman AK, Awang K, Rahman MM, Shilpi JA, Jahan R, Islam E (2015). Ethnomedicinal survey of various communities residing in Garo Hills of Durgapur, Bangladesh. J Ethnobiol Ethnomed.

[9] Khisha T, Karim R, Chowdhury SR, Banoo R (2012). Ethnomedical studies of chakma communities of Chittagong hill tracts, Bangladesh. Bangladesh J Pharmacol.

[10] Uddin SB, Faruque MO, Talukder S. A survey of traditional health remedies of the Chakma Indigenous community of Rangamati District, Bangladesh. J Plant Sci Res. 2014;1.

[11] Rahmatullah M, Hossan MS, Hanif A, Roy P, Jahan R, Khan M, Chowdhury MH, Rahman T (2009). Ethnomedicinal applications of plants by the traditional healers of the Marma tribe of Naikhongchhari, Bandarban district, Bangladesh. Adv Nat Appl Sci.

[12] Sajib NH, Uddin S (2013). Medico-botanical studies of Sandwip island in Chittagong, Bangladesh. Bangl J Plant Taxon.

[13] Biswas A, Bari M, Roy M, Bhadra S (2010). Inherited folk pharmaceutical knowledge of tribal people in the Chittagong hill tracts, Bangladesh. Indian J Tradit Know.

[14] Kadir MF, Sayeed MSB, Mia M (2012). Ethnopharmacological survey of medicinal plants used by indigenous and tribal people in Rangamati, Bangladesh. J Ethnopharmacol.

[15] Islam A, Siddik AB, Hanee U, Guha A, Zaman F, Mokarroma U, Zahan H, Jabber S, Naurin S, Kabir H (2015). Ethnomedicinal practices among a Tripura community in rangamati district, Bangladesh. Int J Pharm Pharm Sci.

[16] Banglapedia. National Encyclopedia of Bangladesh. In: Asiatic Society of Bangladesh. Dhaka: Asiatic Society of Bangladesh; 2003.

[17] Rao R. Methods and techniques in ethnobotanical study and research: some basic consideration. In: Jain SK, editor. Methods and Approaches in Ethnobotany - Society of Ethnobotanists, Lucknow. 1989. p. 13-23.

[18] Given DR, Harris W. Techniques and methods of ethnobotany: as an aid to the study, evaluation, conservation and sustainable use of biodiversity. London: Commonwealth Secretariat Publications; 1994.

[19] Alexiades MN, Sheldon JW. Selected guidelines for ethnobotanical research: a field manual. New York: New York Botanical Garden; 1996.

[20] Martin GJ: Ethnobotany: a methods manual*.* London, UK: Earthscan; 1995.

[21] Heinrich M, Ankli A, Frei B, Weimann C, Sticher O (1998). Medicinal plants in Mexico: healers’ consensus and cultural importance. Soc Sci Med.

[22] Logan MH: Informant consensus: a new approach for identifying potentially effective medicinal plants. Plants in indigenous medicine and diet: biobehavioral approaches 1986, 91.

[23] Heinrich M (2000). Ethnobotany and its role in drug development. Phytother Res.

[24] González-Tejero MR, Casares-Porcel M, Sánchez-Rojas CP, Ramiro-Gutiérrez JM, Molero-Mesa J, Pieroni A, Giusti ME, Censorii E, de Pasquale C, Della A (2008). Medicinal plants in the Mediterranean area: synthesis of the results of the project Rubia. J Ethnopharmacol.

[25] Weckerle CS, de Boer HJ, Puri RK, van Andel T, Bussmann RW, Leonti M (2018). Recommended standards for conducting and reporting ethnopharmacological field studies. J Ethnopharmacol.

[26] Eissa TAF, Palomino OM, Carretero ME, Gómez-Serranillos MP (2014). Ethnopharmacological study of medicinal plants used in the treatment of CNS disorders in Sinai Peninsula, Egypt. J Ethnopharmacol.

[27] Kadir MF, Sayeed MSB, Mia M (2013). Ethnopharmacological survey of medicinal plants used by traditional healers in Bangladesh for gastrointestinal disorders. J Ethnopharmacol.

[28] Ong HG, Kim Y-D (2014). Quantitative ethnobotanical study of the medicinal plants used by the Ati Negrito indigenous group in Guimaras island, Philippines. J Ethnopharmacol.

[29] Sadat-Hosseini M, Farajpour M, Boroomand N, Solaimani-Sardou F (2017). Ethnopharmacological studies of indigenous medicinal plants in the south of Kerman, Iran. J Ethnopharmacol.

[30] Zheng X-l, Xing F-W (2009). Ethnobotanical study on medicinal plants around Mt.Yinggeling, Hainan Island, China. J Ethnopharmacol.

[31] IUCN: The IUCN Red List of Threatened Species. 2017. http://www.iucnredlist.org. Accessed 7 Feb 2018.

[32] Marles RJ, Farnsworth NR (1995). Antidiabetic plants and their active constituents. Phytomedicine.

[33] Suleiman MHA (2015). An ethnobotanical survey of medicinal plants used by communities of Northern Kordofan region, Sudan. J Ethnopharmacol.

[34] Uddin SB, Ratna RS, Faruque MO (2013). Ethnobotanical study on medicinal plants of Rakhaing Indigenous Community of Cox's Bazar District of Bangladesh. J Pharmacogn Phytochem.

[35] Faruque M, Uddin S (2014). Ethnomedicinal study of the Marma community of Bandarban district of Bangladesh. Acad J Med Plant.

[36] Rahman KR, Faruque MO, Uddin SB, Hossen I (2016). Ethnomedicinal knowledge among the local community of Atwari Upazilla of Panchagarh District, Bangladesh. Int J Trop Agric.

[37] Giday M, Asfaw Z, Woldu Z (2010). Ethnomedicinal study of plants used by Sheko ethnic group of Ethiopia. J Ethnopharmacol.

[38] Telefo P, Lienou L, Yemele M, Lemfack M, Mouokeu C, Goka C, Tagne S, Moundipa F (2011). Ethnopharmacological survey of plants used for the treatment of female infertility in Baham, Cameroon. J Ethnopharmacol.

[39] Sarker MN, Mahin AA, Munira S, Akter S, Parvin S, Malek I, Hossain S, Rahmatullah M (2013). Ethnomedicinal plants of the Pankho community of Bilaichari Union in Rangamati district, Bangladesh. Am-Eur J Sustain Agr.

[40] Uddin MZ, Hassan MA (2014). Determination of informant consensus factor of ethnomedicinal plants used in Kalenga forest, Bangladesh. Bangl J Plant Taxon.

[41] Malla B, Gauchan DP, Chhetri RB (2015). An ethnobotanical study of medicinal plants used by ethnic people in Parbat district of western Nepal. J Ethnopharmacol.

[42] Islam MK, Saha S, Mahmud I, Mohamad K, Awang K, Jamal Uddin S, Rahman MM, Shilpi JA (2014). An ethnobotanical study of medicinal plants used by tribal and native people of Madhupur forest area, Bangladesh. J Ethnopharmacol.

[43] Manandhar NP (1995). A survey of medicinal plants of Jajarkot district, Nepal. J Ethnopharmacol.

[44] Ocvirk S, Kistler M, Khan S, Talukder SH, Hauner H (2013). Traditional medicinal plants used for the treatment of diabetes in rural and urban areas of Dhaka, Bangladesh–an ethnobotanical survey. J Ethnobiol Ethnomed.

[45] Rajakumar N, Shivanna MB (2009). Ethno-medicinal application of plants in the eastern region of Shimoga district, Karnataka, India. J Ethnopharmacol.

[46] Das PR, Islam MT, Mostafa MN, Rahmatullah M (2013). Ethnomedicinal plants of the Bauri tribal community of Moulvibazar district, Bangladesh. Anc Sci Life.

[47] Rahman A (2013). Ethno-medicinal investigation on ethnic community in the northern region of Bangladesh. Am J Life Sci.

[48] Pal DC, Jain SK: Tribal medicine*.* Naya Prokash 206, Bidhan Sarani, Calcutta, India; 1998.

[49] Akter S, Nipu AH, Chyti HN, Das PR, Islam MT, Rahmatullah M (2014). Ethnomedicinal plants of the Shing tribe of Moulvibazar district, Bangladesh. World J Pharm Pharmaceut Sci.

[50] Kamal Z, Bairage JJ, Moniruzzaman DP, Islam M, Faruque M, Islam MR, Paul PK, Islam MA, Rahmatullah M (2014). Ethnomedicinal practices of a folk medicinal practitioner in Pabna district, Bangladesh. World J Pharm Pharmaceut Sci.

[51] Rahman A (2015). Ethnomedicinal survey of angiosperm plants used by Santal tribe of Joypurhat District, Bangladesh. Int J Adv Res.

[52] Wahab A, Roy S, Habib A, Bhuiyan M, Roy P, Khan M, Azad AK, Rahmatullah M (2013). Ethnomedicinal wisdom of a Tonchongya tribal healer practicing in Rangamati district, Bangladesh. Am-Eur J Sustain Agr.

[53] Sarker B, Akther F, Sifa U, Jahan I, Sarker M, Chakma S, Podder P, Khatun Z, Rahmatullah M (2012). Ethnomedicinal investigations among the Sigibe clan of the Khumi tribe of Thanchi sub-district in Bandarban district of Bangladesh. Am-Eur J Sustain Agr.

[54] Hossan MS, Roy P, Seraj S, Mou SM, Monalisa MN, Jahan S, Khan T, Swarna A, Jahan R, Rahmatullah M (2012). Ethnomedicinal knowledge among the Tongchongya tribal community of Roangchaari Upazila of Bandarban district, Bangladesh. Am-Eur J Sustain Agr.

[55] Azam MNK, Ahmed MN, Rahman MM, Rahmatullah M (2013). Ethnomedicines used by the Oraon and Gor tribes of Sylhet district, Bangladesh. Am-Eur J Sustain Agr.

[56] Akhter J, Khatun R, Akter S, Akter S, Munni T, Malek I, Rahmatullah M (2016). Ethnomedicinal practices in Natore district, Bangladesh. World J Pharm Pharmaceut Sci.

[57] Azad A, Mahmud MR, Parvin A, Chakrabortty A, Akter F, Moury SI, Anny IP, Tarannom SR, Joy S, Chowdhury S (2014). Ethnomedicinal surveys in two Mouzas of Kurigram district, Bangladesh. World J Pharm Pharmaceut Sci.

[58] Kosalge SB, Fursule RA (2009). Investigation of ethnomedicinal claims of some plants used by tribals of Satpuda Hills in India. J Ethnopharmacol.

[59] Singh A, Singh PK (2009). An ethnobotanical study of medicinal plants in Chandauli District of Uttar Pradesh, India. J Ethnopharmacol.

[60] Ijaz F, Iqbal Z, Rahman IU, Alam J, Khan SM, Shah GM, Khan K, Afzal A (2016). Investigation of traditional medicinal floral knowledge of Sarban Hills, Abbottabad, KP, Pakistan. J Ethnopharmacol.

[61] Aziz MA, Khan AH, Adnan M, Izatullah I (2017). Traditional uses of medicinal plants reported by the indigenous communities and local herbal practitioners of Bajaur agency, federally administrated tribal areas, Pakistan. J Ethnopharmacol.

[62] Khumbongmayum AD, Khan M, Tripathi R (2005). Ethnomedicinal plants in the sacred groves of Manipur. Indian J Tradit Know.

[63] Rai PK, Lalramnghinglova H (2010). Ethnomedicinal plant resources of Mizoram, India: implication of traditional knowledge in health care system. Ethnobot Leaflets.

[64] Sharma HK, Chhangte L, Dolui AK (2001). Traditional medicinal plants in Mizoram, India. Fitoterapia.

[65] Rai PK, Lalramnghinglova H (2010). Lesser known ethnomedicinal plants of Mizoram, North East India: an Indo-Burma hotspot region. J Med Plants Res.

[66] Lalfakzuala R, Kayang H, Lalramnghinglova H (2015). Ethnobotanical usages of plants in western Mizoram. Indian J Tradit Know.

[67] Shil S, Choudhury MD, Das S (2014). Indigenous knowledge of medicinal plants used by the Reang tribe of Tripura state of India. J Ethnopharmacol.

[68] Sen S, Chakraborty R, De B, Devanna N (2011). An ethnobotanical survey of medicinal plants used by ethnic people in west and south district of Tripura, India. J Forest Res.

[69] Sajem AL, Gosai K (2006). Traditional use of medicinal plants by the Jaintia tribes in North Cachar Hills district of Assam, Northeast India. J Ethnobiol Ethnomed.

[70] McGaw L, Jäger A, Van Staden J, Eloff J (2002). Isolation of β-asarone, an antibacterial and anthelmintic compound, from Acorus calamus in South Africa. S Afr J Bot.

[71] Faruque O, Uddin S (2011). Ethnodiversity of medicinal plants used by Tripura community of Hazarikhil in Chittagong district of Bangladesh. J Taxon Biodiv Res.

[72] Islam MK, Saha S, Mahmud I, Mohamad K, Awang K, Uddin SJ, Rahman MM, Shilpi JA (2014). An ethnobotanical study of medicinal plants used by tribal and native people of Madhupur forest area, Bangladesh. J Ethnopharmacol.

[73] Gangadevi V, Muthumary J (2008). Taxol, an anticancer drug produced by an endophytic fungus Bartalinia robillardoides Tassi, isolated from a medicinal plant, *Aegle marmelos* Correa ex Roxb. World J Microbiol Biotechnol.

[74] Mishra BB, Singh DD, Kishore N, Tiwari VK, Tripathi V (2010). Antifungal constituents isolated from the seeds of Aegle marmelos. Phytochemistry.

[75] Phuwapraisirisan P, Puksasook T, Jong-Aramruang J, Kokpol U (2008). Phenylethyl cinnamides: a new series of α-glucosidase inhibitors from the leaves of Aegle marmelos. Bioorg Med Chem Lett.

[76] Zhang XF, Wang HM, Song YL, Nie LH, Wang LF, Liu B, Shen PP, Liu Y (2006). Isolation, structure elucidation, antioxidative and immunomodulatory properties of two novel dihydrocoumarins from Aloe vera. Bioorg Med Chem Lett.

[77] Lawrence R, Tripathi P, Jeyakumar E (2009). Isolation, purification and evaluation of antibacterial agents from Aloe vera. Braz J Microbiol.

[78] Lee KH, Kim JH, Lim DS, Kim CH (2000). Anti-leukaemic and anti-mutagenic effects of Di (2-ethylhexyl) phthalate isolated from aloe vera Linne. J Pharm Pharmacol.

[79] Yenjit P, Issarakraisila M, Intana W, Chantrapromma K (2010). Fungicidal activity of compounds extracted from the pericarp of Areca catechu against Colletotrichum gloeosporioides in vitro and in mango fruit. Postharvest Biol Tech.

[80] Uchino K, Matsuo T, Iwamoto M, Tonosaki Y, Fukuchi A (1988). New 5′-nucleotidase inhibitors, NPF-86IA, NPF-86IB, NPF-86IIA, and NPF-86IIB from Areca catechu; part I. Isolation and biological properties. Planta Med.

[81] Kraus W, Bokel M, Bruhn A, Cramer R, Klaiber I, Klenk A, Nagl G, Pöhnl H, Sadlo H, Vogler B (1987). Structure determination by NMR of azadirachtin and related compounds from Azadirachta indica A. Juss (Meliaceae). Tetrahedron.

[82] Kabir MH, Hasan N, Rahman MM, Rahman MA, Khan JA, Hoque NT, Bhuiyan MRQ, Mou SM, Jahan R, Rahmatullah M (2014). A survey of medicinal plants used by the Deb barma clan of the Tripura tribe of Moulvibazar district, Bangladesh. J Ethnobiol Ethnomed.

[83] Saratha V, Pillai SI, Subramanian S (2011). Isolation and characterization of lupeol, a triterpenoid from Calotropis gigantea latex. Int J Pharm Sci Rev Res.

[84] Sen S, Sahu NP, Mahato SB (1992). Flavonol glycosides from Calotropis gigantea. Phytochemistry.

[85] Shaker KH, Morsy N, Zinecker H, Imhoff JF, Schneider B (2010). Secondary metabolites from Calotropis procera (Aiton). Phytochem Lett.

[86] Ibrahim SR, Mohamed GA, Shaala LA, Banuls LMY, Van Goietsenoven G, Kiss R, Youssef DT (2012). New ursane-type triterpenes from the root bark of Calotropis procera. Phytochem Lett.

[87] Daisy P, Balasubramanian K, Rajalakshmi M, Eliza J, Selvaraj J (2010). Insulin mimetic impact of Catechin isolated from Cassia fistula on the glucose oxidation and molecular mechanisms of glucose uptake on streptozotocin-induced diabetic Wistar rats. Phytomedicine.

[88] Duraipandiyan V, Ignacimuthu S (2010). Antifungal activity of rhein isolated from *Cassia fistula* L. flower. Webmed Central Pharmacol.

[89] Azad A, Mahmud MR, Parvin A, Chakrabortty A, Akter F, Moury SI, Anny IP, Tarannom SR, Joy S, Chowdhury S (2014). Medicinal plants of a Santal tribal healer in Dinajpur district, Bangladesh. World J Pharm Pharmaceut Sci.

[90] Nhiem NX, Tai BH, Quang TH, Van Kiem P, Van Minh C, Nam NH, Kim J-H, Im L-R, Lee Y-M, Kim YH (2011). A new ursane-type triterpenoid glycoside from Centella asiatica leaves modulates the production of nitric oxide and secretion of TNF-α in activated RAW 264.7 cells. Bioorg Med Chem Lett.

[91] Rahmatullah M, Mollik AH, Khatun A, Jahan R, Chowdhury AR, Seraj S, Hossain MS, Nasrin D, Khatun Z (2010). A survey on the use of medicinal plants by folk medicinal practitioners in five villages of Boalia sub-district, Rajshahi district, Bangladesh. Adv Nat Appl Sci.

[92] Revathy S, Elumalai S, Antony MB. Isolation, purification and identification of curcuminoids from turmeric (*Curcuma longa* L.) by column chromatography. J Exp sci. 2011;2(7):21-5.

[93] He X-G, Lin L-Z, Lian L-Z, Lindenmaier M (1998). Liquid chromatography–electrospray mass spectrometric analysis of curcuminoids and sesquiterpenoids in turmeric (Curcuma longa). J Chromatogr A.

[94] Rahman M, Hossan MY, Aziz N, Mostafa MN, Mahmud MS, Islam MF, Seraj S, Rahmatullah M. Home remedies of the Teli clan of the Telegu tribe of Maulvibazar district, Bangladesh. Am-Eur J Sustain Agr. 2013:290–5.

[95] Jha DK, Panda L, Lavanya P, Ramaiah S, Anbarasu A (2012). Detection and confirmation of alkaloids in leaves of Justicia adhatoda and bioinformatics approach to elicit its anti-tuberculosis activity. Appl Biochem Biotechnol.

[96] Rahmatullah M, Mollik MAH, Harun-or-Rashid M, Tanzin R, Ghosh KC, Rahman H, Alam J, Faruque MO, Hasan MM, Jahan R. A comparative analysis of medicinal plants used by folk medicinal healers in villages adjoining the Ghaghot, Bangali and Padma Rivers of Bangladesh. Am-Eur J Sustain Agr. 2010:70–86.

[97] Patil R, Patil R, Ahirwar B, Ahirwar D (2011). Isolation and characterization of anti-diabetic component (bioactivity—guided fractionation) from Ocimum sanctum L.(Lamiaceae) aerial part. Asian Pac J Trop Med.

[98] Kumaran A, Karunakaran RJ (2006). Nitric oxide radical scavenging active components from Phyllanthus emblica L. Plant Foods Hum Nutr.

[99] Sadhu SK, Khatun A, Phattanawasin P, Ohtsuki T, Ishibashi M (2007). Lignan glycosides and flavonoids from Saraca asoca with antioxidant activity. J Nat Med.

[100] Varghese GK, Bose LV, Habtemariam S (2013). Antidiabetic components of Cassia alata leaves: identification through α-glucosidase inhibition studies. Pharm Biol.

[101] Rahaman M, Hasan AM, Ali M, Ali M (2006). A flavone from the leaves of Cassia alata. Bangladesh J Sci Ind Res.

[102] Cuervo AC, Blunden G, Patel AV (1991). Chlorogenone and neochlorogenone from the unripe fruits of Solanum torvum. Phytochemistry.

[103] Lu Y, Luo J, Huang X, Kong L (2009). Four new steroidal glycosides from Solanum torvum and their cytotoxic activities. Steroids.

[104] Sudjaroen Y, Haubner R, Würtele G, Hull W, Erben G, Spiegelhalder B, Changbumrung S, Bartsch H, Owen R (2005). Isolation and structure elucidation of phenolic antioxidants from tamarind (Tamarindus indica L.) seeds and pericarp. Food Chem Toxicol.

[105] Wong K, Tan C, Chow C, Chee S (1998). Volatile constituents of the fruit of Tamarindus indica L. J Essent Oil Res.

[106] Karim S, Rahman M, Shahid SB, Malek I, Rahman A, Jahan S, Jahan FI, Rahmatullah M. Medicinal plants used by the folk medicinal practitioners of Bangladesh: a randomized survey in a village of Narayanganj district. Am-Eur J Sustain Agr. 2011:405–15.

[107] Reddy DB, Reddy T, Jyotsna G, Sharan S, Priya N, Lakshmipathi V, Reddanna P (2009). Chebulagic acid, a COX–LOX dual inhibitor isolated from the fruits of Terminalia chebula Retz., induces apoptosis in COLO-205 cell line. J Ethnopharmacol.

[108] Manosroi A, Jantrawut P, Akazawa H, Akihisa T, Manosroi J (2010). Biological activities of phenolic compounds isolated from galls of Terminalia chebula Retz.(Combretaceae). Nat Prod Res.

[109] Mukti M, Ahmed A, Chowdhury S, Khatun Z, Bhuiyan P, Debnath K, Rahmatullah M (2012). Medicinal plant formulations of Kavirajes in several areas of Faridpur and Rajbari districts, Bangladesh. Am-Eur J Sustain Agr.

[110] Chen C-C, Rosen RT, Ho C-T (1986). Chromatographic analyses of isomeric shogaol compounds derived from isolated gingerol compounds of ginger (Zingiber officinale Roscoe). J Chromatogr A.

[111] Hasan SA, Uddin M, KNU H, Das A, Tabassum N, Hossain R, Mahal MJ, Rahmatullah M. Ethnomedicinal plants of two village folk medicinal practitioners in Rajshahi district, Bangladesh: comparison of their folk medicinal uses with Ayurvedic uses. Am-Eur J Sustain Agr. 2014:10–20.

